# Genomes of the Mouse Collaborative Cross

**DOI:** 10.1534/genetics.116.198838

**Published:** 2017-06-06

**Authors:** Anuj Srivastava, Andrew P. Morgan, Maya L. Najarian, Vishal Kumar Sarsani, J. Sebastian Sigmon, John R. Shorter, Anwica Kashfeen, Rachel C. McMullan, Lucy H. Williams, Paola Giusti-Rodríguez, Martin T. Ferris, Patrick Sullivan, Pablo Hock, Darla R. Miller, Timothy A. Bell, Leonard McMillan, Gary A. Churchill, Fernando Pardo-Manuel de Villena

**Affiliations:** * The Jackson Laboratory, Bar Harbor, Maine 04609; † Department of Genetics, University of North Carolina, Chapel Hill, North Carolina 27599; ‡ Lineberger Comprehensive Cancer Center, University of North Carolina, Chapel Hill, North Carolina 27599; § Curriculum of Bioinformatics and Computational Biology, University of North Carolina, Chapel Hill, North Carolina 27599; ** Department of Computer Science, University of North Carolina, Chapel Hill, North Carolina 27599; †† Curriculum of Genetics and Molecular Biology, University of North Carolina, Chapel Hill, North Carolina 27599

**Keywords:** whole genome sequence, drift, selection, genetic variants, multiparental populations, MPP

## Abstract

The Collaborative Cross (CC) is a multiparent panel of recombinant inbred (RI) mouse strains derived from eight founder laboratory strains. RI panels are popular because of their long-term genetic stability, which enhances reproducibility and integration of data collected across time and conditions. Characterization of their genomes can be a community effort, reducing the burden on individual users. Here we present the genomes of the CC strains using two complementary approaches as a resource to improve power and interpretation of genetic experiments. Our study also provides a cautionary tale regarding the limitations imposed by such basic biological processes as mutation and selection. A distinct advantage of inbred panels is that genotyping only needs to be performed on the panel, not on each individual mouse. The initial CC genome data were haplotype reconstructions based on dense genotyping of the most recent common ancestors (MRCAs) of each strain followed by imputation from the genome sequence of the corresponding founder inbred strain. The MRCA resource captured segregating regions in strains that were not fully inbred, but it had limited resolution in the transition regions between founder haplotypes, and there was uncertainty about founder assignment in regions of limited diversity. Here we report the whole genome sequence of 69 CC strains generated by paired-end short reads at 30× coverage of a single male per strain. Sequencing leads to a substantial improvement in the fine structure and completeness of the genomes of the CC. Both MRCAs and sequenced samples show a significant reduction in the genome-wide haplotype frequencies from two wild-derived strains, CAST/EiJ and PWK/PhJ. In addition, analysis of the evolution of the patterns of heterozygosity indicates that selection against three wild-derived founder strains played a significant role in shaping the genomes of the CC. The sequencing resource provides the first description of tens of thousands of new genetic variants introduced by mutation and drift in the CC genomes. We estimate that new SNP mutations are accumulating in each CC strain at a rate of 2.4 ± 0.4 per gigabase per generation. The fixation of new mutations by genetic drift has introduced thousands of new variants into the CC strains. The majority of these mutations are novel compared to currently sequenced laboratory stocks and wild mice, and some are predicted to alter gene function. Approximately one-third of the CC inbred strains have acquired large deletions (>10 kb) many of which overlap known coding genes and functional elements. The sequence of these mice is a critical resource to CC users, increases threefold the number of mouse inbred strain genomes available publicly, and provides insight into the effect of mutation and drift on common resources.

GENETIC reference populations derived from multiple parents, or multiparent populations (MPPs), have become popular in a wide variety of model organisms (Churchill *et al.* 2004; Kover *et al.* 2009; Huang *et al.* 2011; King *et al.* 2012; Bouchet *et al.* 2017; Cubillos *et al.* 2017; King and Long 2017; Mangandi *et al.* 2017; Najarro *et al.* 2017; Raghavan *et al.* 2017: Stanley *et al.* 2017; Tisné *et al.* 2017). The Collaborative Cross (CC) (Supplemental Material, Table S1 lists all abbreviations used throughout this manuscript) is a mouse MPP derived from eight inbred strains that was initially conceived as a mapping population for complex traits and as a platform for integration of phenotypic data across a reproducible set of variable genotypes (our approach to infuse genetics into the field of systems biology) (Threadgill *et al.* 2002; Churchill *et al.* 2004). The CC project first had to overcome an assortment of logistic and financial hurdles (Chesler *et al.* 2008; Iraqi *et al.* 2008; Morahan *et al.* 2008; Collaborative Cross Consortium 2012). More importantly, our goals had to face the unrelenting resistance of biology that led to an extraordinary rate of extinction among the hundreds of CC lines that were initially set up (Shorter *et al.* 2017). Despite these challenges, the CC has been used successfully in both complex trait mapping and systems genetics (Aylor *et al.* 2011; Kelada *et al.* 2012; Ferris *et al.* 2013; Gralinski *et al.* 2015, 2017; Green *et al.* 2017). All of these studies point to the expansion of the phenotypic range previously described in laboratory mice and its continuous distribution for many traits. In addition, these studies reported the breakdown of phenotypic correlations previously thought to be hardwired. It has become evident that CC strains represent a rich source of murine models for human diseases that do not exist or are underrepresented in standard laboratory mice (Rogala *et al.* 2014). A timely example of convergence of the main three uses of the CC is provided by a recent study on the effect of genetic variation on Ebola virus susceptibility (Rasmussen *et al.* 2014).

The CC has been a catalyst for the development of many new resources and tools for mouse genetics. For example, the Diversity Outbred (DO) population is a popular outbred MPP derived from a subset of incompletely inbred CC lines (Svenson *et al.* 2012). Tracking the inbreeding and founder contribution in the CC was the motivation behind the development of widely used genotyping platforms such as the Mouse Diversity Array (Yang *et al.* 2009) and the several iterations of the Mouse Universal Genotyping Array (MUGA) (Collaborative Cross Consortium 2012; Morgan *et al.* 2015). The CC was the seed for the development of a wide variety of analytic and informatics tools and methods from haplotype mosaic reconstruction, to improvement in the interpretation of genotyping arrays, to genotype imputation of mouse populations, and to new databases (Mott *et al.* 2000; Fu *et al.* 2012; Welsh *et al.* 2013; Gatti *et al.* 2014; Oreper *et al.* 2017). Finally, mouse MPPs in general and the CC and DO in particular were an impetus to sequencing, characterization, and *de novo* assembly of the genomes of an ever-expanding collection of mouse laboratory strains (Keane *et al.* 2011; Yalcin *et al.* 2011; Wong *et al.* 2012; Ananda *et al.* 2014; Doran *et al.* 2016; Morgan *et al.* 2016a). Collectively these contributions have opened new and exciting avenues to answer key long-standing biological questions (Chick *et al.* 2016; Morgan *et al.* 2017).

Previous studies have reported the genotypes of incompletely inbred CC lines, typically one mouse per line (Aylor *et al.* 2011; Kelada *et al.* 2012; Ferris *et al.* 2013; Gralinski *et al.* 2015). More importantly, in 2012 a large collaborative study reported the CC genome architecture based on genotypes of a single mouse from >300 CC lines using the first iteration of the MUGA family of arrays (Collaborative Cross Consortium 2012). This study included CC lines from all three breeding sites that contributed to the CC population [Oak Ridge National Laboratory (ORNL), United States; Tel Aviv University (Tau), Israel; Geniad LLC (Geni), Australia]. Shortly after, CC distribution centers were setup with a uniform set of rules that aimed to ensure the genetic integrity of each strain, expedite inbreeding progress, and facilitate unrestricted access to the CC by researchers independent of their affiliation to the CC project (Welsh *et al.* 2012). A key decision was to release each CC strain for public use once it reached a minimum level of inbreeding (typically, at least 90% of the genome fixed for a founder haplotype in that strain). This step requires genotyping of the obligate most recent common ancestors (MRCAs) within each CC strain that has reached the inbreeding threshold. The genome of this set of distributable CC strains is considerably better characterized than the initial CC lines because for each CC strain, we genotyped at least two MRCAs using the second generation, higher-density MegaMUGA genotyping platform (Welsh *et al.* 2012). Whole genome sequences are available for the CC strains based on imputation from founder strains sequences (Keane *et al.* 2011) in each of the haplotype segments identified through genotyping of the MRCAs (Huang *et al.* 2014). Although genotypes, haplotype reconstructions, and imputed genomes based on the MRCAs have been released publicly and are used in published studies, no global analysis of the actual genomes of the distributable CC strains exists.

Here we report our analyses of the genomes of 69 CC strains based on two complementary sets of genetic data. First we describe the CC population based on the MegaMUGA genotyping of their MRCAs and second, on whole genome sequence of a single male descended from these MRCAs. Among our main reasons to sequence the CC strains were the desire to increase the spatial resolution of the recombination breakpoints between consecutive haplotypes, to resolve haplotype origin in regions of identity by descent (IBD) that could not be confidently assigned by MUGA genotyping, to identify new mutations introduced by drift during the generation of the CC, and to determine the role of selection in shaping the genome of the CC strains as they reach complete inbred status. The results presented here will improve the power and resolution of the CC as a mapping population, help to explain the emergence of disease phenotypes (CC strains that are phenotypic outliers) in CC strains, and guide the interpretation of findings in the CC and related mouse reference populations.

## Materials and Methods

### Mice

All CC mice were obtained from the Systems Genetics Core Facility at the University of North Carolina (UNC) (Welsh *et al.* 2012). Prior to their relocation to UNC, CC strains were generated and bred at Tel Aviv University in Israel (Iraqi *et al.* 2008), Geniad LLC in Australia (Morahan *et al.* 2008), and Oak Ridge National Laboratory in the United States (Chesler *et al.* 2008). CC mice can be obtained from the Systems Genetics Core Facility at UNC (http://csbio.unc.edu/CCstatus/index.py).

### DNA isolation

Whole genomic DNA for genotyping in the MUGA family of arrays was isolated from tail using Qiagen Gentra Puregene or DNeasy Blood and Tissue kits according to the manufacturer’s instructions. High molecular weight DNA for whole genome sequencing was isolated from a large tail piece by standard phenol chloroform extraction from a single male obtained from the Systems Genetics Core Facility (Welsh *et al.* 2012).

### Genotyping

All genome-wide genotyping was performed using the first, second, and third iterations of the Mouse Universal Genotyping Array, MUGA, MegaMUGA, and GigaMUGA (GeneSeek, Lincoln, NE) (Collaborative Cross Consortium 2012; Morgan *et al.* 2015). Genotypes were called using Illumina BeadStudio (Illumina, Carlsbad, CA) and processed with Argyle (Morgan 2015). All genotypes are available at Zenodo (https://doi.org/10.5281/zenodo.377036).

### Sequencing

Samples were sequenced at the UNC High Throughput Sequencing Facility or at the New York Genome Center (NYGC). The genomic DNA from eight CC strains (Table S2) was submitted to the UNC High Throughput Sequencing Facility at a concentration of at least 40 ng/μl in 60 μl. Genomic DNAs were sheared by ultrasonication and the resulting fragments were size selected to target size 350 bp using a PippinPrep system. The UNC High Throughput Sequencing Facility generated sequencing libraries using Kapa (Kapa Biosystems) DNA Library Preparation Kits for Illumina sequencing. Each CC sample was run on its own lane of a HiSeq4000 (Illumina), and generated 150-bp paired end reads.

For the remaining 61 strains (Table S2), genomic DNA was submitted to The Jackson Laboratory High Throughput Sequencing Core Facility. DNAs were sheared using the E220 Focused-ultrasonicator (Covaris, Woburn, MA). The Jackson Laboratory core generated whole genome libraries using the Kapa Hyper Prep Kit for Illumina Sequencing (Kapa Biosystems), targeting an insert size of 300 bp using magnetic bead-based size selection. Libraries were pooled and sequenced on the HiSeqX (Illumina) at the NYGC, and generated 150-bp paired end reads.

A single C57BL/6J mouse was obtained from The Jackson Laboratory’s pedigreed stock and was sequenced at ∼30× coverage at NYGC as described for the 69 CC strains.

### Burrows–Wheeler transforms

We built multistring Burrows–Wheeler transforms (msBWTs) of the raw fastq sequencing lanes from the 69 sequenced CC samples, the fastq files of the seven founder strains sequenced by the Sanger Institute (Keane *et al.* 2011), and a C57BL/6J sample provided by The Jackson Laboratory. The msBWTs were constructed using a hybrid combination of ropeBWT (Li 2014) and the msBWT merge algorithm (Holt and McMillan 2014) as described at https://github.com/holtjma/msbwt/wiki. The msBWTs are a lossless compressed form of the raw, sequenced reads that can be efficiently queried to find number of occurrences of and/or the associated read fragments containing any specified subsequence or k-mer. The msBWTs were used to efficiently genotype each of the sequenced samples, in less time than a short-read alignment followed by variant calling. The msBWTs were also used to do targeted genome assemblies in regions to resolve the exact boundaries of genomic deletions, and to resolve the boundaries of recombination events.

### Haplotype reconstruction and 36-state probabilities

All individual MRCA samples were genotyped at 70,000+ markers using the second generation MUGA genotyping platform, MegaMUGA (Morgan *et al.* 2015). The raw hybridization intensities were used to infer the posterior probabilities of all 36 founder genotype combinations (8 homozygous and 28 potential heterozygous diplotypes) at each marker using the forward–backward hidden Markov model (HMM) previously reported (Fu *et al.* 2012). Individual sample probabilities were then conservatively combined to account for regions where MRCAs exhibited different alleles. These joint genotype probabilities for each CC strain are available at http://csbio.unc.edu/CCstatus/CCGenomes.

The genetic variants ascertained by Sanger Institute’s Mouse Genomes Project (MGP) were used to infer the founder origin for the genomes of all sequenced samples (Keane *et al.* 2011). A set of 42.2 million informative variants between the eight CC founders were extracted from the May 2015, version 5 release of the MGP SNPs and indels variant call format (VCF) file. From this set we selected 31.3 million variants that were biallelic among the CC founder strains and with no other variant within 12 bases. We excluded all *Y* chromosome variants, due to uncertainties in the sex of the samples sequenced by the Sanger Institute. We replaced these with 42 *Y* chromosome variants from the Affymetrix Mouse Diversity Array (Yang *et al.* 2009, 2011) that are known to segregate between CC founder males and generate no-calls in female samples. In addition, the msBWTs were used to ascertain 22 additional *Y* variants. The resulting 64 marker set (available at http://csbio.unc.edu/CCstatus/CCGenomes) was sufficient to distinguish each of the eight founders in the presence of errors and reduced hemizygous coverage.

A set of 125.2 million (31.3 million variants times two alleles for both the forward and reverse strand) virtual 25-base genotyping probe sequences were created for the forward and reverse complement sequences centered about both reference and alternate variants. We queried the msBWTs of all sequenced CC samples and msBWTs of the eight founder strains (seven sequenced in the MGP, and the sequenced C57BL/6J). We then filtered the variant set to remove unusually high and low probe-sequence counts occurring in any of the sequenced samples, further reducing the total set of variants used for inferring the founder origin to 19.9 million. The number of occurrences for each remaining probe sequence was used to infer the founder genotypes and haplotypes of each CC sample via the HMM.

The emission probabilities used in the HMM models the likelihood that a given set of probe counts could have been generated by the set of expected founder variants within each 5-kb nonoverlapping windows in each of 36 possible founder state combinations (8 homozygous + 28 heterozygous) assuming a 1% sequencing error rate. We forced a neutral emission model (1/36 probability for each genotype state) in 5-kb windows for which the haplotype of the founder MGP sample was not the highest probability as expected. Finally, the transition probabilities modeled the likelihood of recombination between adjacent 5-kb windows. We solved the posterior marginals of each genotype state in all 5-kb genomic windows using a forward–backward algorithm. A similar approach, but with only 8 genotype states instead of 36, was used to infer the posterior marginals of the *X* chromosome, given that these are all male samples.

Finally, diplotypes were constructed by detecting runs of the maximal inferred posterior probabilities along genomes. Adjacent genotype states were phased to minimize the total number of haplotype transitions. These inferred haplotype reconstructions and posterior genotype probabilities for every 5 kb-genomic window are available at www.csbio.unc.edu/CCstatus/CCGenomes.

We were able to further refine recombination breakpoints using the msBWTs. We queried the sequenced read fragments from each sample for every nonoverlapping 59-mer from the reference genome within a window of 25 kb centered around the transitions inferred by the HMM. By comparing the read-coverage patterns surrounding the recombination to other sequenced samples with the expected founder on either side of the recombination, we were able in most cases to establish the recombination boundary to the nearest informative variant whether or not it was annotated, a SNP, an indel, or a microsatellite.

### Founder haplotype contribution

We tested for differential contribution of founder strains to heterozygous *vs.* homozygous regions of the genome as follows: For each sequenced individual, the observed fraction of the autosomes assigned to each of the 36 possible diplotype states was computed. Those 36 values were collapsed to 8 values corresponding to the contribution of each founder haplotype and a single value corresponding to the total heterozygous fraction. The expected fraction of the genome in each of the 28 heterozygous states was calculated as the product of the marginal contribution of the corresponding founder haplotypes, holding the total heterozygous fraction of the genome constant at the observed value. We finally calculated the quantity: log_2_(observed het fraction/expected het fraction) for each of the 28 heterozygous states, adding a small pseudocount to both the numerator and the denominator so that the logarithm is always defined.

### Mapping of unplaced sequences

The current mouse reference genome assembly (mm 10/GRCm38.p5) consists of 22 chromosome-level components (*1–19*, *X*, *Y*, and *M*) and 44 unplaced components. Of these, 22 have been assigned to a chromosome but not localized to a specific position, and 22 are completely unplaced (Table S3). Their size ranges from 1976 bp (GenBank ID JH584295) to 953,012 bp (JH584299). These sequences consist almost entirely of either repetitive sequences associated with centromeres or telomeres or fragments of segmental duplication clusters. We exploited the fact that such sequences frequently vary in copy number to genetically map them in the CC. Copy number for each CC strain was calculated by estimating the normalized depth of coverage over each component. Each component was mapped as an independent quantitative trait using founder haplotype probabilities estimated from line MRCAs on the grid defined by the MegaMUGA array. QTL scans were performed under an additive model with no polygenic term using R/qtl2. Peaks with LOD score >10 were counted as true hits and are reported as the position of the most highly associated SNP from MegaMUGA.

### Whole genome sequence analysis workflow

We mapped sequence reads in fastq format to the mouse reference genome (University of California Santa Cruz build-mm 10 with *1–19*, *X*, *Y*, and *M* and unplaced loci) using Burrows–Wheeler aligner (BWA) (0.7.9a) aligner (Li 2013) with default parameters. The alignment was sorted by coordinates and converted to binary alignment map (BAM) format by Picard (http://picard.sourceforge.net) SortSam utility. The Picard MarkDuplicates (1.95) module was used to remove duplicates from data.

The BAM file, after removal of duplicates, was then input to Genome Analysis Tool Kit (GATK) HaplotypeCaller (3.4–0) (McKenna *et al.* 2010; DePristo *et al.* 2011) with default parameters to call SNP and small indel variants for each CC strain. We merged variant call results using vcf-merge utility of vcftools (0.1.12a) (Danecek *et al.* 2011). We also performed joint variant calling across 69 samples by GATK HaplotypeCaller in gVCF mode. Variant flags were assigned using SnpSift utilities (4.1g) (Cingolani *et al.* 2012) to identify (a) fixed differences from the reference that are present across all CC strains, (b) variants previously reported (Keane *et al.* 2011), (c) variants in regions of simple sequence repeats (SSRs), (d) variants that overlap with CpG loci in any of the founder strains, (e) complex/multinucleotide polymorphism (MNP) variants, and (f) variants that are present in joint variant calling. Finally, variant annotation was performed by snpEFF (4.1g) (Cingolani *et al.* 2012) and the highest impact annotation for any variant was retained. Genome-wide haplotype reconstructions described above were used to assign a founder haplotype to each variant. We uploaded all ∼53.6 million variant calls into a SQLite database for further analysis.

Three classes of variants derived from this database were further used for analysis and were selected according to the following criteria: high-quality (HQ) variants have read depth ≥15 in at least one animal (for chromosomes *X* and *Y* ≥8); alternate allele frequency of ≥0.2; not fixed; SSR; complex/MNP; locus present in joint calling and alt allele matches the alt allele in joint calling; high-quality homozygous (HQHom) variants meet the above criteria and in addition they must have reference allele depth <2 in at least one animal; private variants meet the HQHom criterion; they occur in exactly one animal; the haplotype of an animal on which the variant occurs should be shared by at least one other animal that carries the reference allele at that site; and finally, the variant should not be present in founder strains (Keane *et al.* 2011). We note that for the majority of samples ≥80% of genome was covered at ≥15× depth (Table S2).

### Pseudogenome creation

We created the pseudogenome for each animal using g2gtools (https://github.com/churchill-lab/g2gtools) utilities (vcf2chain, patch, and transform). To create pseudogenomes, we used variants homozygous for alternate alleles and have read depth of ≥15. Approximately, 6 million SNPs were incorporated in each animal. Finally, we adjusted the annotation and extracted the genes, exons, and transcriptome by g2gtools-convert and g2gtools-extract utilities, respectively. All the pseudogenomes are available through the European Nucleotide Archive (ENA) accession no. PRJEB14673.

### Deletion discovery

We counted read coverage in bins of size 1 kb spanning the entire mouse genome (excluding “N” regions). The distribution of read counts in each bin was calculated using the bedmap module of BEDOPS (v2.4.2) (Neph *et al.* 2012) and a custom script. We filtered the binned read counts to identify runs of consecutive bins with low read counts (<4 reads per kb) and low variance [median absolute deviation (MAD) <6].

Twenty-three large (>1 kb) *de novo* deletions present in only one CC strain were selected for additional characterization. Using the msBWTs, we assembled sequence across the deletion breakpoints and aligned it to the reference genome to determine the precise boundaries of the deletion. Flanking sequences were classified as unique or repetitive using annotations from the Ensembl genome browser (v87/GRCm38.p5) (Yates *et al.* 2016). In the former case, we used the msBWT to identify sequence reads that span the deletion breakpoint and determined the presence of sequence similarity. The gene and regulatory content of the deletion was lifted from mouse Ensembl genome browser.

### Structural variation analysis

The Picard MarkDuplicates processed alignment files were used as an input to Genome STRiP 2.0 (Handsaker *et al.* 2015) for copy number variation (CNV) discovery. Ploidy map and reference files (prerequisite for Genome STRiP) were constructed from mouse reference genome (mm10). We used the tiling window size of 1 kbp and minimum refined length of 500 bp to detect the variants ≥500 kbp. We further selected the sites with evidence of polymorphism and built the raw CNV call set.

### Deletion validation

Primers were designed to amplify both wild-type and “deletion” alleles for three deletions: CC026/GeniUnc (chr17:57 Mb), CC007/Unc (chr13:53 Mb), and CC055/TauUnc (chr3:133 Mb). For each deletion, a forward primer was designed to anneal proximal to the start of the deletion and two reverse primers were designed, one within the deleted region and one just distal of the deleted region (Table S4). PCR were performed with all three primers to score the presence and absence of the deletion and wild-type alleles. The reactions were performed under the following conditions: 95° for 2 min, 35 cycles of 95°, 55°, 72° for 30 sec each, followed by a 72° hold for 7 min. PCR reactions were visualized on 2% agarose gels with ethidium bromide. Each PCR reaction contained 1 μl crude genomic DNA, 2 μl 5× PCR buffer (Promega, Madison, WI), 1 μl dNTP mix (2.5 mM each), 0.3 μl of each primer, 0.1 μl Taq (GoTaq DNA polymerase, Promega) and H_2_O for a total of 10-μl reaction volume.

### *Npnt* gene expression levels

Brain hemispheres were collected from 13 CC055/TauUnc mice (six females and seven males). Samples were genotyped as described above and classified as wt/wt (*n* = 4), wt/del (*n* = 5), or del/del (*n* = 4) at *Npnt*. Brain hemispheres were pulverized using a BioPulverizer unit (BioSpec Products, Bartlesville, OK). Total RNA was extracted from ∼25 mg of powdered brain hemisphere tissue using an automated bead-based capture technology (Maxwell 16 Tissue LEV Total RNA Purification Kit, AS1220; Promega). Purified mRNA was evaluated for quality and quantity by Nanodrop Spectrophotometer (Thermo Fisher Scientific, Waltham, MA). For each sample, complementary DNA (cDNA) was synthesized using 200 ng of starting RNA according to the manufacturer’s protocol (SuperScript III First-Strand Synthesis System, 18080051; Thermo Fisher Scientific).

We used two commercially available Taqman qPCR assays for *Npnt* (Mm00473794_m1 located outside the deletion and Mm01316817_m1 located inside the deletion; Life Technologies, Carlsbad, CA) to estimate gene expression levels inside and outside of the deletion region. For a reference assay, we used the *Hprt* gene expression assay (Mm03024075_m1, Life Technologies) to calibrate the amplification curve. Assays were performed according to the manufacturer’s protocol on an ABI StepOne Plus Real-Time PCR System (Life Technologies). Assays were done in duplicate for each sample and *Npnt* assay. Using ABI CopyCaller v2.0 software on default settings, we determined the cycle thresholds (Ct) for each assay. Samples with failed gene expression reactions were removed from the analysis. For each sample, the relative cycle threshold (ΔCt) was calculated as ΔCt = Ct^target^ − Ct^reference^. The ΔCt value represents the relative gene expression level of the target gene on the log scale. The mean ΔCt value for each biological replicate was calculated from the technical replicates. Unpaired *t*-tests were used to test for differences in ΔCt values between CC055/TauUnc mice with different genotypes at *Npnt*.

### Data availability

A wide range of complementary data resources were used to analyze the CC genomes. Second generation MegaMUGA genotypes (∼70,000 markers) are available for the MRCAs of each CC strain. From these genotypes, we inferred all possible founder origin combinations at each marker genotype (8 inbred and 28 heterozygous combinations for autosomes and 8 founder for hemizygous chromosomes and mitochondria), which, hereafter we refer to as “36-state probabilities” founder probabilities at each marker of each individual MRCA. The founder probabilities from the MRCA set of a CC strain were conservatively merged into a composite MRCA 36-state probability to estimate residual heterozygosity (evidence of multiple alleles at any marker between different MRCA samples was declared heterozygous). The founder probabilities of MRCA samples and their composites are summarized by selecting the maximum-likelihood genotype at each locus and allocating its alleles across two pseudophased haplotypes such that the number of founder transitions were minimized, resulting in a “hapfile.” All hapfiles were tested to be consistent with the original genotypes. Statistics on the number of recombinations, the fraction of founder contribution to each CC strain, and chromosome ideograms are derived from these hapfiles.

A similar inference pipeline was applied to the 69 sequenced samples, which were also genotyped using the third generation GigaMUGA (∼140,000 markers). The hapfiles and 36-state founder probabilities of different genotyping platforms can be directly compared. We also chose to infer the founder probabilities from the sequenced data using the msBWTs so that the results (36-state probabilities and hapfiles) were compatible, but at much higher resolution, with the genotyped samples.

A set of three samples from CC018/Unc were later genotyped using the first generation MUGA platform (∼7,000 markers), to assess how representative the sequenced sample is of its CC line. Once more, we generated 36-state founder inferences and hapfiles to allow us to compare results ascertained by different platforms and technologies.

The genomic founder mosaics, as represented by the hapfiles, were used to partition the CC strain genomes according to their origin for calling new and private mutations. The resulting browser extensible data (BED) and VCF files are provided.

All of the founder inference-related data resources: genotypes, the 36-state probabilities of samples and MRCA composites, hapfiles, chromosome ideograms, and msBWTs for each sequenced CC line, are available online (http://csbio.unc.edu/CCstatus/CCGenomes).

The ENA accession PRJEB14673 provides access to the following files:

1. CC_69_Samples_vcf_merge.db: SQLlite database file.
2. Genomestrip_raw.v*cf*.gz: genome strip unfiltered calls.
3. Joint_69_flagged.tab: VCFfile obtained by joint haplotype variant caller.
4. Merged_69_flagged.tab: VCF file merged from single sample calling.
5. pseudoGenomes.tar.gz: Pseudo genome files.

Zenodo accession no. 377036 provides access to the following files:

1. fastq_filelist: list of fastq filenames deposited with ENA.
2. bam_file_list: list of bam filenames deposited with ENA.
3. CC_69_samples-1 kb_haplo.bed: read coverage in kilobase bins, used for deletion analysis.
4. bin_creator.py: code to create read depth files.
5. count_calculator.sh: shell script to create read depth files.
6. CNV_Analysis_1k.R: analysis of read depth data.
7. CCStrains.csv: summary information about CC strains.
8. Private_Variants.csv: list of 28,000 private variants.
9. PrivateVariants.R: analysis of private variants data.
10. Hap files based on GigaMUGA genotypes, whole genome sequence, and MRCAs.
11. The 36-state probabilities for 69 MRCAs.
12. Genotype files for 69 sequenced samples in GigaMUGA, all MRCAs, and three CC018/Unc samples genotyped in MUGA.

## Results

### Nomenclature and status of the CC population

The concept of the CC was first laid out in 2002 (Threadgill *et al.* 2002) followed by a white paper (Churchill *et al.* 2004) and a series of proof-of-principle experiments (Fouldes-Mathes *et al.* 2011; Aylor *et al.* 2011; Durrant *et al.* 2011; Kelada *et al.* 2012; Ferris *et al.* 2013; Gralinski *et al.* 2015). In 2012 the CC Consortium provided the first overview of the genomes of the CC lines (Collaborative Cross Consortium 2012). However, unfettered access to the CC population has only recently become a reality. This reflects the decision not to release a CC strain until it had reached a minimum level of inbreeding (typically >90% of the genome fixed by haplotype) and the need to establish distribution centers to ensure a common level of genetic QC (Welsh *et al.* 2012). Determination of the level of inbreeding relied on genotyping of select individuals within each pedigree and identification of obligate ancestors (MRCAs) of each strain that jointly fulfilled the requirement for the level of inbreeding. Once a strain is deemed distributable, it is renamed as CCxxx/yyy where “x” are consecutive numbers and “y” are several letter identifiers of the breeding site where the CC strain was initiated and currently maintained (Unc, Tau, and Geni).

Here we report two sets of genetic data, high-density genotypes of MRCAs and whole genome sequence for a single male mouse, for 69 CC strains that have reached the required level of inbreeding and are available to the community (Table S2). These strains originate from all three initial breeding sites with 30 strains derived from the UNC population (Chesler *et al.* 2008), 24 strains from the Geniad population (Morahan *et al.* 2008), and 15 strains from the Tau population (Iraqi *et al.* 2008). Two strains, CC0051/TauUnc and CC0059/TauUnc, are not fully independent as they derive from a single lineage and were separated and bred independently for at least 15 generations (Collaborative Cross Consortium 2012) (Table S2).

On average, there are 2.9 MRCAs per strain (range 2–9) and these MRCAs have undergone on average 19 generations of inbreeding (range 14–36) (Table S2). All MRCAs were genotyped on MegaMUGA (Morgan *et al.* 2015) and their founder origin was inferred. Whole genome sequence was generated from a single male per CC strain that had undergone on average 7 more generations of inbreeding beyond the corresponding MRCAs (range 1–14 generations) (Table S2). These mice were also genotyped with GigaMUGA for comparison purposes. All genotypes and founder inferences are available online (http://csbio.unc.edu/CCstatus/CCGenomes).

**Figure 1:**
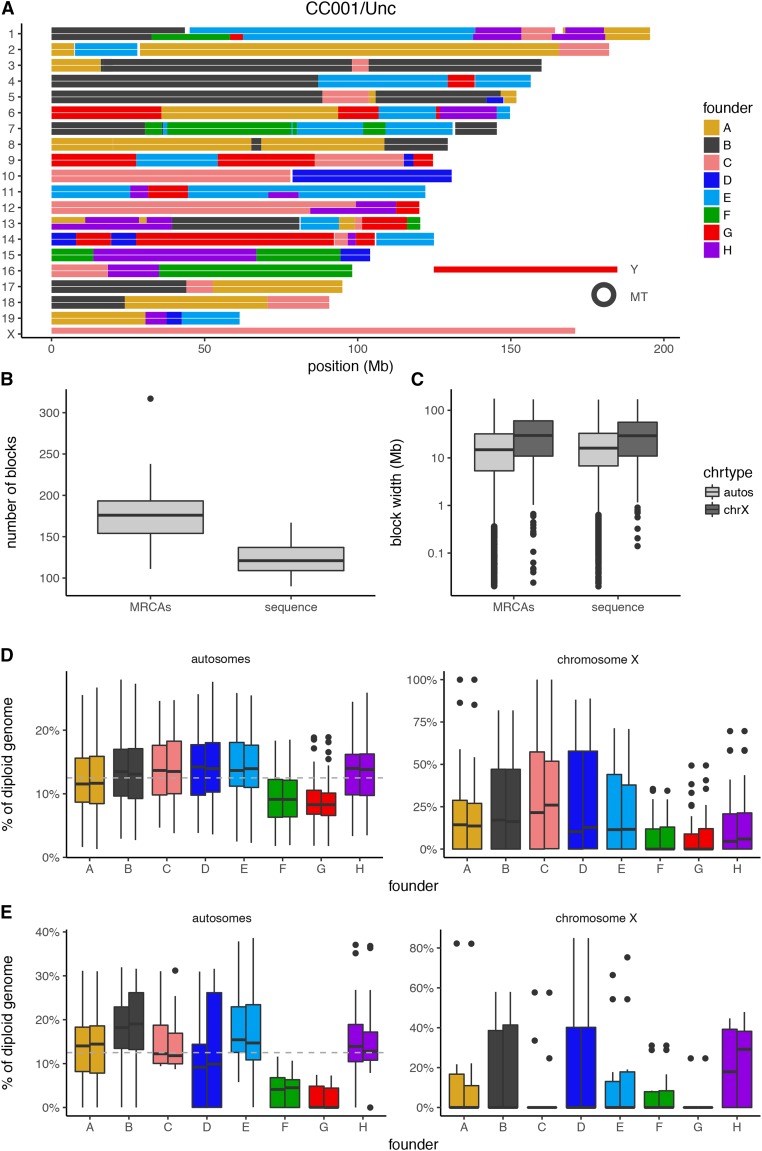
The CC genomes. In all figures we use the following colors and letter codes to represent the eight founder strains of the CC: A/J, yellow (A); C57BL/6J, gray (B); 129S1/SvImJ, pink (C); NOD/ShiLtJ, dark blue (D); NZO/HlLtJ, light blue (E); CAST/EiJ, green (F); PWK/PhJ, red (G); and WSB/EiJ, purple (H). (A) Haplotype mosaic for the sequenced representative of the CC001/Unc strain. (B) Number of haplotype blocks identified in the MRCA and sequenced samples. (C) Distribution of haplotype block size in MRCAs and sequenced samples in log scale. (D) Founder contribution to the genomes of CC strains with all eight founders. Autosomes are shown in the left panel and chromosome *X* in the right. Within a panel and founder strain the left boxes represent MRCAs and the right, the sequenced sample. (E) Founder contribution to the genomes of CC strains with missing founders. Founder contribution to chromosome *X*. Autosomes are shown in the left panel and chromosome *X* in the right. Within a panel and founder strain the left boxes represent MRCAs and the right, the sequenced sample.

On average, 2.5 years separate MRCAs from the sequenced sample from the same CC strain (range 53–1726 days) (Table S2). Two additional years have passed since the birth of the sequenced sample (range 368–1198 days). We reconstructed the haplotypes of MRCAs and sequenced strains (Figure 1A).

### CC genomes of MCRAs and sequenced sample

We combined the MRCAs as previously described by Welsh *et al.* (2012) to generate a strain haplotype that retains each possible founder haplotype present locally in any of the MRCAs. Based on the MRCA haplotypes, we determined the number of founders present in each strain (Table S2). Eight CC strains have only six founders, 5 have seven founders, and the remaining 56 have contributions from all eight founders (Figure S1 and Table S2). PWK/PhJ is missing in 8 CC strains, CAST/EiJ is missing in 5 strains, NOD/ShiLtJ in 4, and A/J, C57BL/6J, NZO/HILtJ, and WSB/EiJ are missing in 1 strain each (Table S2). These results are consistent with the previous report of missing founders in the genomic fraction of CC strains (Collaborative Cross Consortium 2012). Missing founder status is also consistent between MRCAs and sequenced samples.

After haplotype reconstruction, we determined the number of haplotype blocks and their size distribution in both the MRCAs and sequenced CC populations (Figure 1, B and C). The median number of haplotype blocks is 176 in the MRCAs (range 111–317) and 121 in the sequenced samples (range 90–167) (Figure 1B). Overall, these values are in line with the expectations for the CC (Broman 2012) and the difference between MRCAs and sequenced samples is due to the presence of multiple samples in each set of MRCAs and the reduction in heterozygosity in the sequenced sample (see below). The analysis of the haplotype block size was performed independently in the autosomes and chromosome *X* (Figure 1C). The median block size in the autosomes is 15 Mb in the MRCAs and 16.3 Mb in the sequenced samples, but there is a wide variation in size (range 20 kb–176 Mb in the MRCAs and 20 kb–171 Mb in the sequenced samples).

**Figure 2:**
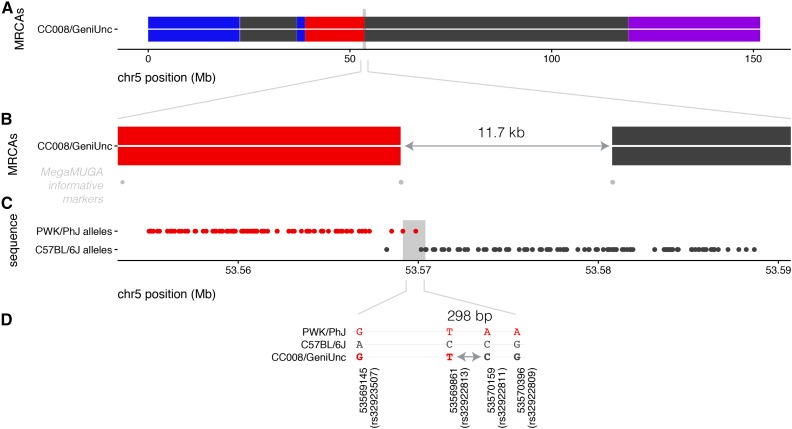
Sequencing improves haplotype assignment in recombination intervals. (A) Haplotype reconstruction for chromosome *5* from MRCAs of CC044/GeniUnc. The focal recombination event is indicated by a gray box. (B) Zoomed-in view of recombination interval, showing the flanking informative markers from the MegaMUGA genotyping array. Haplotype assignment in the MRCAs is uncertain over 11.7 kb. (C) Alleles in the sequenced CC044/GeniUnc male shared with PWK/PhJ (top track) or C57BL/6J (bottom track); inferred recombination interval is indicated by a gray box. (D) Genotypes at informative SNPs between PWK/PhJ and C57BL/6J reduce the recombination interval to 298 bp, between rs32922813 and rs32922811.

Use of the sequence data results in a reduction of haplotype uncertainty at the recombination breakpoints compared to the MRCAs (Figure S2). The length of recombination intervals is reduced 12-fold for 82% of the crossover events (median size of 1.6 kb *vs.* 19.4 kb for sequenced and MRCAs, respectively) (Figure S3). In the best-case scenario, breakpoints are resolved to recombination intervals spanned by short sequence reads. In the example shown in Figure 2, the recombination interval in the sequenced sample is 2.5% as long as the one detected by genotyping in the MRCAs (298 bp *vs.* 11.7 kb, respectively). Given that many of these recombination intervals span known genes, this should also increase power and precision of mapping studies. However, 18% of the recombination intervals remain poorly resolved and in fact some increase in size in the sequenced samples (Figure S4). Larger intervals are associated with IBD between founder inbred strains and poorly characterized regions of the genome.

On average, residual heterozygosity in the CC strains at the MCRA level spans 8.04% of the autosomal genome (range 0.5–15%, Table S2). As expected, the mice sequenced are significantly more inbred than the union of their MRCA progenitors (average 2.89%, range 0–9.27%), but the correlation between percentage of the genome fixed and the number of generations between MRCA and sequenced animal is weak (Pearson’s *r* = 0.24) as is the correlation between the level of inbreeding in the MRCA and in the sequenced mouse (Pearson’s *r* = −0.30). Based on the single male sequenced, 19 strains have <1% residual heterozygosity (but note that 6 of the 19 strains have one or more missing founders). Heterozygosity is found across the genome and is more prevalent in larger autosomes and less so on chromosome *X* (Figure S4), reflecting the expected negative correlation with recombination (Broman 2012). Finally, strains with all eight founders tend to be more heterozygous than strains with missing founders: 8.54% in strains with eight founders, 4.74% in strains with seven founders, and 6.27% in strains with six founders in both MRCAs and sequenced samples (Table S2).

The founder contributions to the 69 CC strains are highly consistent between MRCAs and the sequenced samples (Table 1). However, across both sample sets the founder contribution is not uniform, with the contributions of both CAST/EiJ and PWK/PhJ to the autosomal genomes being significantly lower than the expected 12.5% (8.55 and 7.41%, respectively). At first glance, this could be explained by the number of times that a founder strain is missing (Table S2). However, further analysis restricted to the CC strains with contributions from all eight founder strains shows that unequal contribution persists with lower contributions of PWK/PhJ (8.63%) and CAST/EiJ (9.58%).

**Table 1:** Founder contribution to the genomes of the CC strains

Population	Generation	Chr	A/J	C57BL/6J	129S1/SvImJ	NOD/ShiLtJ	NZO/HlLtJ	CAST/EiJ	PWK/PhJ	WSB/EiJ
All	MRCAs	Aut	12.84	14.54	14.18	13.75	14.93	8.55	7.41	13.80
With eight founders only	MRCAs	Aut	12.41	13.59	13.93	14.21	14.48	9.58	8.63	13.16
All	Sequenced	Aut	12.60	14.61	14.34	13.87	14.83	8.53	7.45	13.78
With eight founders only	Sequenced	Aut	12.26	13.49	14.23	14.49	14.27	9.45	8.63	13.18
All	Sequenced	*X*	10.75	16.58	19.73	19.81	12.44	5.06	4.30	11.33
With eight founders only	Sequenced	*X*	10.63	16.81	22.84	18.99	12.35	4.58	4.85	8.95
With eight founders only	Sequenced	*Y*	6	8	7	11	8	8	5	3
With missing founders	Sequenced	*Y*	1	2	3	1	5	0	1	0
With eight founders only	Sequenced	*M*	19*	15	7	19*	6	2	2	5
With missing founders	Sequenced	*M*	7*	0	2	7*	2	1	0	1

For the autosomes and the *X* chromosome the table shows the percentage of contribution of each founder. For chromosome *Y* and mitochondria the table shows the number of CC strains in each haplogroup. *, the mitochondria of A/J and NOD/ShiLtJ cannot be distinguished by sequencing so the total number of CC strains sharing these haplotypes are shown in both columns.

Whole genome sequencing of CC males offered the opportunity to improve the founder assignment for two regions of the genome, the mitochondria and the *Y* chromosome, in which the MUGA arrays have limited power to discriminate between CC founders (Chesler *et al.* 2016). As summarized in Table 1, the founder contributions to the mitochondria and the *Y* chromosome are not even, with lower than expected contribution of CAST/EiJ and PWK/PhJ to the former and of WSB/EiJ to the latter.

### Selection in the CC population

Our analysis of the global contribution of the founder strains shows distortions in the haplotype frequency in the autosomes, sex chromosomes, and mitochondria in strains that have contribution from all eight founder strains. In all cases, the distortion results from the underrepresentation of one or more of the three wild-derived strains, CAST/EiJ, PWK/PhJ, and WSB/EiJ. We conclude that the distortion arises through some form of selection (see *Discussion*). To gain further insight, we plotted the founder frequency along the chromosomes (Figure S5) and the founder frequency of contribution per chromosome (Figure S6). No obvious peaks are present in the autosomes, in contrast with the initial analysis of the CC (Aylor *et al.* 2011; Collaborative Cross Consortium 2012) of strong distortion in favor of the WSB/EiJ allele at the *R2d2* locus (Didion *et al.* 2015). Although the representation of CAST/EiJ and PWK/PhJ varies among chromosomes, it is lower than the expectation of 12.5% on 16 autosomes for each of these two strains. We do not observe any significant association between individual autosomes and founder contribution.

**Figure 3:**
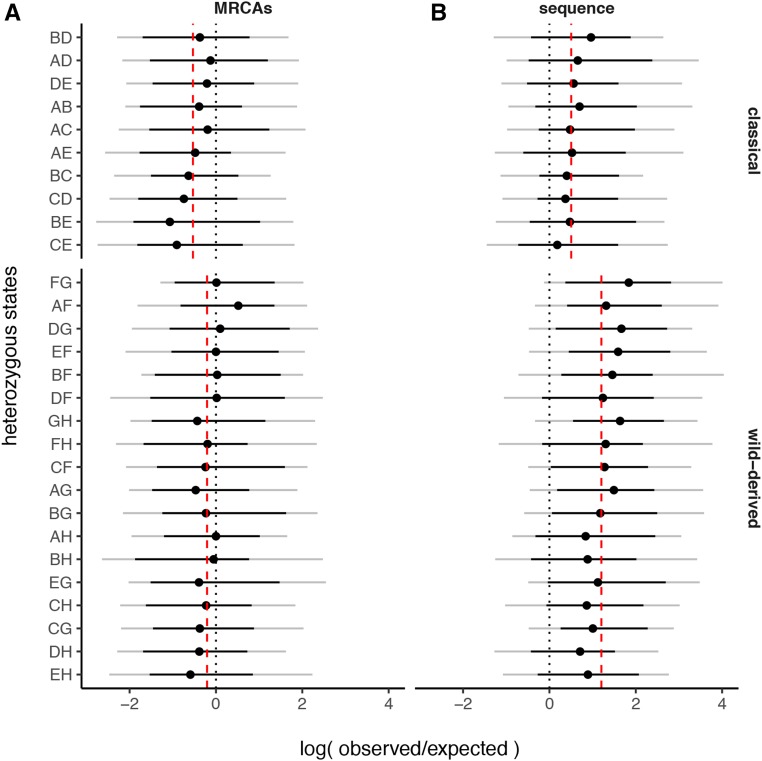
Biased contribution of the CC founders to the residual heterozygosity present in the MRCAs and sequenced samples. The *x*-axis shows log ratio of observed to expected proportion of the genome in each of 28 possible heterozygous states (*y*-axis) across 56 CC strains with all eight founder haplotypes present. Heterozygous states are divided into two classes: those involving classical inbred strains only (top) or those involving at least one wild-derived strain (bottom). Black dotted line gives expected value of the statistic (zero), and gray dashed lines show median value in each panel.

We hypothesized that selection against the wild-derived inbred founders should leave a signature in the regions of residual heterozygosity in the 56 CC strains with contribution from all eight founder strains. To perform this analysis, we calculated the fraction of the genome contributed by each one of the 36 possible diplotype states in both the MRCA and the sequenced sample. We then estimated whether the observed frequency of each one of the 28 heterozygous states is as expected from the overall contribution of the founders to that strain and aggregated data across all 56 CC strains. Interestingly the contribution of the founder strain is not independent of its zygosity status in both cohorts (Wilcoxon rank-sum tests *P* = 8.5 × 10^−3^ *P* = 9.1 × 10^−8^ MRCAs and sequence, respectively) (Figure 3). The three wild-derived strains contribute significantly higher amounts to the heterozygous regions than would be expected from their contribution to the homozygous regions. In fact, the maximum discrepancies between the expected and observed values are found for CAST/EiJ and PWK/PhJ heterozygosity. We conclude that although selection systematically has purged CAST/EiJ and PWK/PhJ from the CC genomes, regions of residual heterozygosity are retained by selection (see *Discussion*).

**Figure 4:**
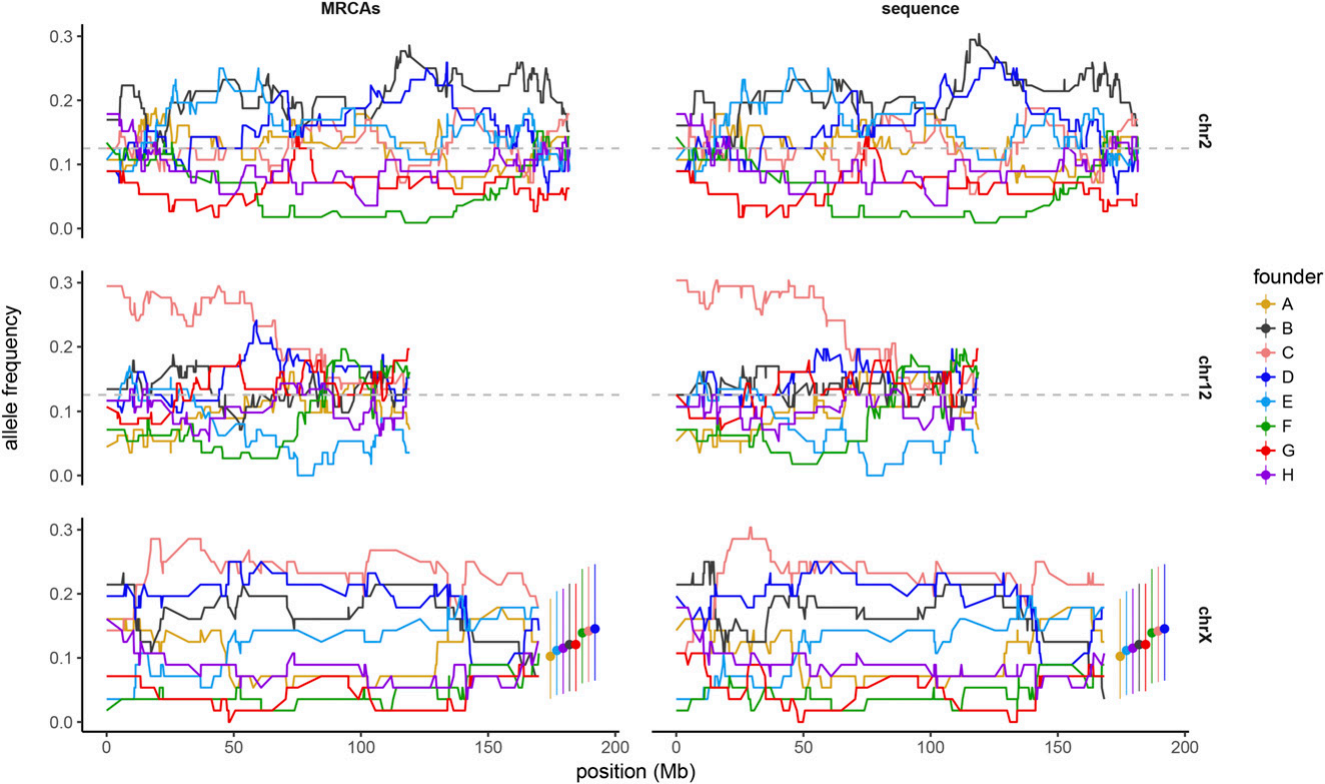
Haplotype frequencies on chromosomes *2*, *12*, and *X* in MRCAs and sequenced samples. The analysis is restricted to the 56 CC strains with all eight founder strains present.

For the *X* chromosome, the expected contribution of each founder strain depends on its position at the start of the breeding funnel (see *Materials and Methods*, Collaborative Cross Consortium 2012). Once these are taken into account, plotting of haplotype frequency along the *X* chromosome reveals strong distortion against PWK/PhJ and CAST/EiJ and mild overrepresentation of the classical 129S1/SvImJ strain (Figure 4).

### Genetic drift in the CC population

To characterize the full scope of genetic variation in the CC, we generated variant calls for SNPs and small indels (primarily <100 bp) for each of the sequenced CC strains. All variants are defined relative to the mm10 reference genome by convention. A typical CC strain yielded 8–9 million candidate variants, which when merged across all 69 strains, generated a total of 53,685,594 variant sites. For each variant call, we recorded total read depth, alternative and reference allele counts, and additional features in a database, which has been deposited to ENA (accession no. PRJEB14673). We recorded whether each variant was previously identified in the founder strains as reported by the Sanger Mouse Genomes project (SNP RELEASE v5: REL-1505-SNPs_Indels). We flagged MNPs (complex/MNP, *n* = 644,668), variants in SSRs (*n* = 3,583,689), variant overlapping with CpG loci in any of the founder strains (CPG, *n* = 4,397,617), variants present in joint calling (JointCall, *n* = 53,200,128), and fixed differences (*n* = 19,955) for which the alternative allele call is present across all 69 CC strains.

Among the 53.7 million total variant calls, 43.3 million meet our HQ criteria (see *Materials and Methods*) and 39.9 million are both high quality and unambiguously homozygous in at least one animal (HQHom). The majority of HQ variants (40.0 million) were previously reported in the founder strain genomes (representing seven founders due to the assumed equivalence of mm10 reference and the C57BL/6J founder strain). The remaining 3.34 million variants are novel.

Among the novel variants, 956,286 occur in multiple CC strains and are always associated with the same haplotype. These likely represent a mix of variants that are not previously reported in the founder strain genomes and variants that distinguish the founder strain animals that were sequenced from the animals that were used to establish the CC strains. In addition, 27,800 HQHom variants were reported in exactly one of the 69 sequenced CC strains and were absent from all other CC strains with the condition that at least one other CC strain shares the same haplotype but carries the reference allele at the variant site. These private variants include *de novo* mutations that arose in the independent CC strain lineages. We caution that variants detected in only one strain are likely to have higher than average calling error rates, especially in wild-derived haplotypes where alignment errors and paralogous variation in gene families may be hard to detect.

The proportion of indels among all 53.7 million variants is 16.8%. This proportions drops to 12.9% of the HQHom variants. The HQHom variants are distributed among the 19 autosomes in proportion to their physical length at a frequency of 13.9 SNPs and 2.1 indels per kilobase. Variant frequencies on the *X* chromosome are lower, with 7.1 SNPs and 1.1 indels per kilobase. The SNP substitutions tabulated for HQHom variants (Table 2) are symmetric with respect to reference and alternative alleles; the transition-to-transversion ratios are all near to their expected value of 2, and 11.0% of SNPs occur at CpG dinucleotides. Functional analysis predicted several of the SNP and indel variants to have high impact on gene function (Table S5).

**Table 2:** Nucleotide substitution rates among the HQHom and private variants

HQHom	ALT	A	C	G	T
REF	A	—	1401759 (4.0%)	5073707 (14.6%)	1455186 (4.2%)
	C	1589874 (4.6%)	—	1072433 (3.1%)	6151991 (17.7%)
	G	6155882 (17.7%)	1072703 (3.1%)	—	1591576 (4.6%)
	T	1460442 (4.2%)	5076553 (14.6%)	1401807 (4.0%)	—
Private	ALT				
		A	C	G	T
REF	A	—	561 (3.8%)	1161 (7.8%)	720 (4.8%)
	C	1183 (7.9%)	—	519 (3.5%)	3323 (22.3%)
	G	3275 (22.0%)	502 (3.4%)	—	1179 (7.9%)
	T	763 (5.1%)	1162 (7.8%)	569 (3.8%)	—

A tabulation of reference (REF) and alternative (ALT) allele at SNPs variant sites for high-quality homozygous (HQHom) variant calls and for SNP variants that occur uniquely in one CC strain (private). The pattern of substitutions shows a high proportion of C-to-T and G-to-A substitutions and a transition–transversion ratio of ∼2.

#### Analysis of private variants:

We identified 27,800 private variants representing the most likely candidates for new mutations that have arisen in the course of inbreeding the CC strains. Among these, 999 are found on the *Y* chromosome and one, a synonymous substitution in *mt-Co2*, is found in the mitochondrial genome. Here we focus on the 26,800 private mutations on the autosomes and *X* chromosome. There are 14,917 SNP variants and 11,883 small indels. Most of the private variants (13,607 SNPs and 9061 indels) are not previously reported in any mouse strains. There are 6.87 private SNPs and 8.33 private indels per megabase on autosomes and 13.78 private SNPs and 15.32 private indels per megabase on the *X* chromosome. The proportion of indels among private variants (44.3%) is substantially higher compared to the HQHom variants. This suggests that errors among the private variant calls are higher for indel calls and may also be higher on the *X* chromosome relative to autosomes. The pattern of nucleotide substitutions among private SNPs (Table 2) and the frequency of variants at CpG sites (*n* = 1343, 9%) are consistent with expectations and similar to the patterns seen in HQHom variants.

**Figure 5:**
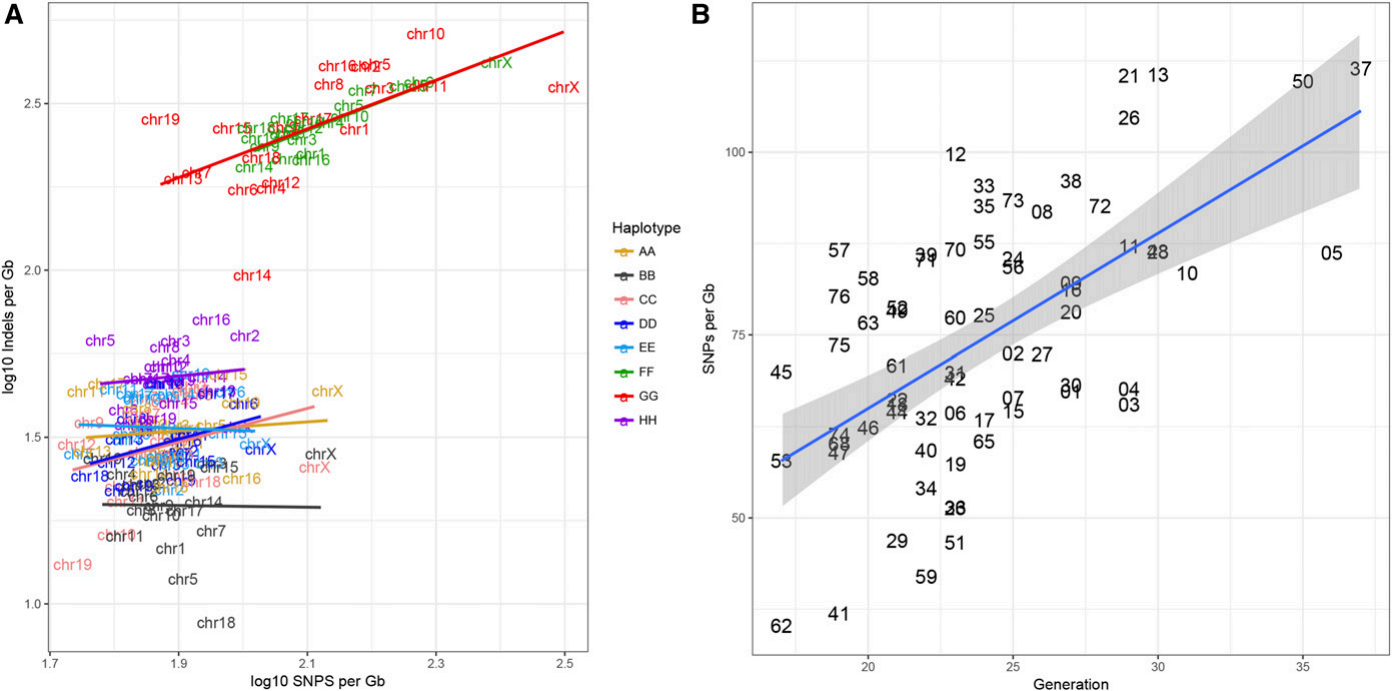
Frequency of private variants in 69 CC strains. (A) The log_10_ frequency per gigabase of SNPs and indels by chromosome (text) and haplotype (color) reveals that wild-derived haplotypes have higher apparent rates of private variation. (B) The strain-specific frequency of SNPs on nonwild autosomal haplotypes was estimated by Poisson regression. The frequency per gigabase of private SNPs increases with the breeding generation of the sequenced animal. The slope of the regression line (2.4 SNPs per gigabase per generation) provides an estimate of the rate of accumulation of new SNPs in the CC strains. Strains are identified by the last two digits of the strain name, *e.g.*, CC002/Unc is indicated as “02” in the figure.

To better understand the genome-wide and haplotype-specific distribution of private variants, we divided the 69 whole genomes into homozygous haplotype blocks. We removed small haplotype blocks (<1 Mb), which represent a tiny fraction (0.03%) of the total genome coverage. The average frequency of private SNPs across all strains, chromosomes, and haplotypes is 0.0867/Mb and the indel frequency is 0.0690/Mb. However, variant call rates associated with PWK/PhJ and CAST/EiJ haplotype blocks are substantially higher than this average (Table S6). The rate of SNP calls is increased by twofold to ∼0.13/Mb and the rate of indels is increased by 10-fold to ∼0.27/Mb. We hypothesize that the excess of variant calls in two wild-derived genomes have resulted from structural differences from the reference genome and paralogous variation. The chromosome and haplotype specific rates of SNP and indels (Figure 5A) are lowest on C57BL/6 haplotypes and moderately increase with genetic distance from the reference strain. Private variant rates on the *X* chromosome (which are expected to have ∼1/2 depth of read coverage compared to autosomes in sequence data obtained from male mice) are generally higher than for autosomes. It is interesting to note that chromosome *14*, which includes an extensive region of introgression from *Mus musculus musculus* into the C57BL/6 genome (Yang *et al.* 2011), has the lowest rates of private variant calls among PWK/PhJ haplotypes in the CC strains.

We used Poisson regression to estimate the frequencies of fixation of new SNP and indel variants by strain, chromosome, and haplotype. Chromosomal differences were primarily due to the autosome *vs. X* contrast and haplotype differences were due to the contrast between CAST/EiJ and PWK/PhJ (wild strains) *vs.* the remaining six founders. The average frequency of private SNP variants on nonwild-derived segments of autosomes is 0.0758/Mb. The frequency of SNP variants is increased 1.66-fold (*P* < 2e−16) on chromosome *X* and is increased 1.70-fold (*P* < 2e−16) in wild-derived haplotypes. The average frequency of private indels is 0.0353/Mb, approximately half the frequency of SNPs. In contrast to SNPs, indel frequency is not substantially different on the *X* chromosome compared to autosomes (1.18-fold, *P* = 1.1e−5). However, the frequency of indels on wild-derived haplotypes is increased 7.77-fold (*P* < 2e−16) compared to the nonwild haplotype blocks. As there is no *a priori* reason to believe that mutation rates are higher in the wild-derived haplotype blocks, we attribute the excess of variants to higher calling error rates in the wild-derived blocks and, to a lesser extent, on the *X* chromosome. To estimate the rate of accumulation of new variants per breeding generation, we obtained estimates of strain-specific variants from the nonwild autosomal haplotype blocks. There is significant variation in the frequency of private SNPs across the CC strains and a significant trend with the breeding generation of the sequenced animal (Figure 5B). The trend corresponds to a rate of SNP accumulation of 2.4 ± 0.4 new SNPs per gigabase per generation (*P* = 8.7e−8). The estimated rate of accumulation of indels is 0.48 ± 0.32 new events per gigabase per generation, but is not statistically significant (*P* = 0.14). The impact of calling errors on the estimation of fixation rates is reflected in the intercept of the regression, because calling errors should occur at random with respect to breeding generation. For SNP variants, the estimated frequency of SNPs at generation zero (*i.e.*, the intercept which is 17.1 SNPs per gigabase) is not statistically different from zero (*P* = 0.084); for indel variants, the intercept (21 indels per gigabase) reaches statistical significance (*P* = 0.0086), consistent with other evidence supporting a higher error rate for calling private indels. Going forward, validation of private variants will provide accurate estimate of calling error rates.

#### Large-scale CNV:

**Figure 6:**
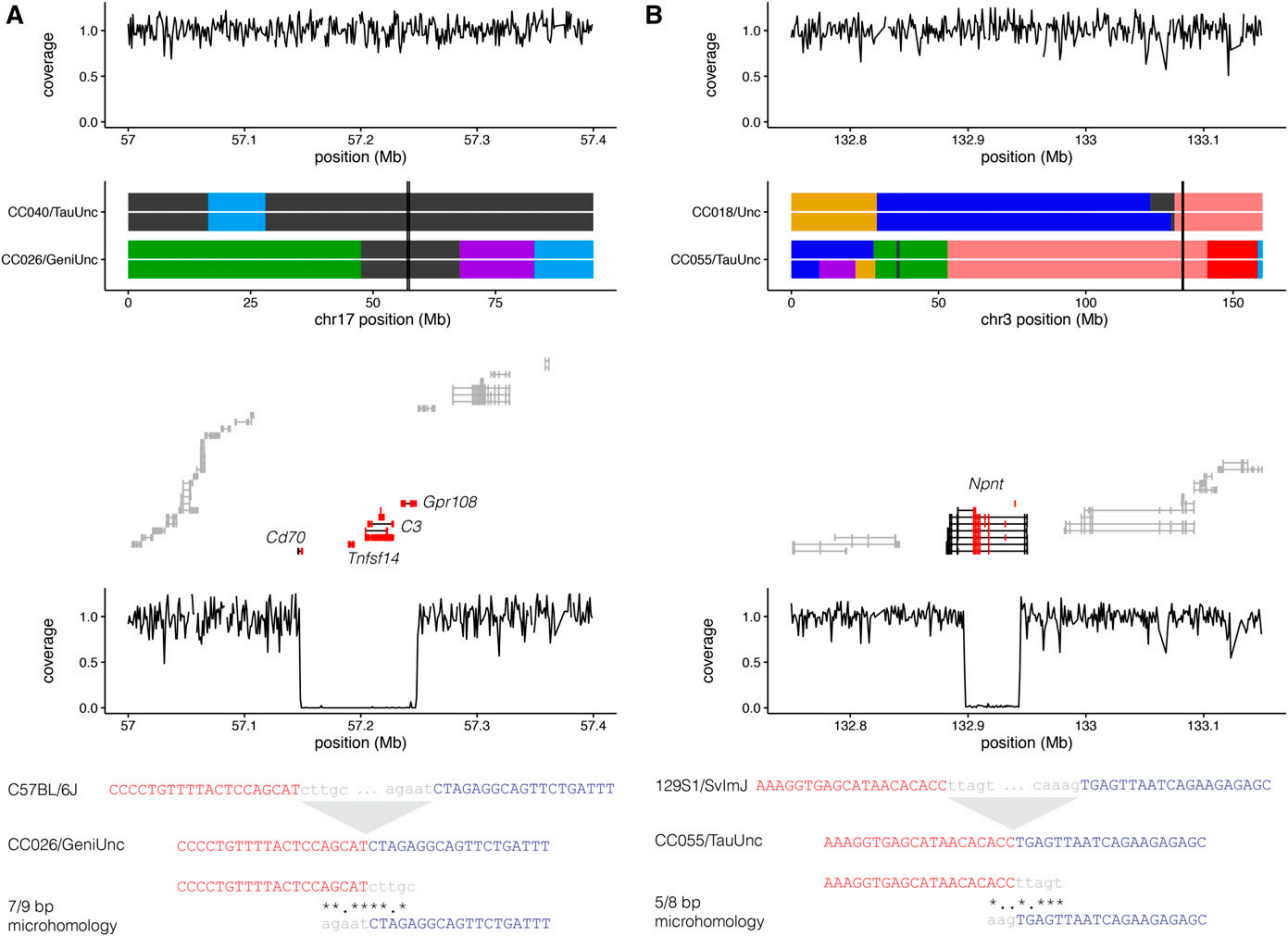
Examples of large private deletions. (A) Deletion on a C57BL/6J haplotype on chromosome *17*: 57 Mb in CC026/GeniUnc is not shared with CC040/Unc, which shares the underlying C57BL/6J haplotype. Top panel shows normalized coverage in whole-genome sequencing (in 1-kb bins) for CC040/Unc; lower panel shows normalized coverage in CC026/GeniUnc. The deletion spans exons (red) from four genes including complement factor gene *C3*. Assembled sequence spanning the deletion shows microhomology over 9 bp at the breakpoint. (B) Deletion on a 129S1/SvImJ haplotype on chromosome *3*: 133 Mb in CC055/TauUnc is not shared with CC018/Unc, which shares the underlying 129S1/SvImJ haplotype. Organization follows that found in A. The deletion spans the middle exons (red) of *Npnt*, which encodes the integrin-binding protein nephronectin.

We identified a total of 26 deletions >1 kb in size, each present in a single CC strain and not previously reported (Table 3). Deletion range from ∼1 to 100 kb in size and several deletions overlap coding genes and annotated functional DNA elements. The availability of multiple CC strains with the same local haplotype that do not carry the corresponding deletions will aid in the functional analysis of these DNA elements. Some deletions are flanked by repeat elements. Figure 6 shows two examples of large *de novo* deletions with predicted deleterious effect in gene function in two different CC strains. Both examples are flanked by microhomology and the breakpoints were resolved using the msBWTs.

**Table 3:** Analysis of 23 selected *de novo* deletions

Strain	Chr	Start	End	Size (kb)	Start (refined with BWT)	End (refined with BWT)	Size (refined with BWT)	Haplotype	Genes	Regulatory Elements	Overlaps with known SV	Notes
CC004	2	116,465,000	116,480,000	15	ND	ND	ND	WSB/EiJ	None	None	Yes	Repeat
CC004	18	75,868,000	75,896,000	28	75,863,985	75,900,551	36,566	C57BL/6J	*Zbtb7c* (intron)	ENSMUSR00000378576, ENSMUSR00000378577, ENSMUSR00000378578	Yes	Unique, splice site
CC006	2	36,341,000	36,366,000	25	ND	ND	ND	PWK/PhJ	None	ENSMUSR00000152903	Yes	Olfactory receptor cluster
CC007	13	53,304,000	53,318,000	14	53,299,892	53,322,383	22,491	NOD/ShiLtJ	None	ENSMUSR00000074496, ENSMUSR00000339964, ENSMUSR00000074498, ENSMUSR00000339965, ENSMUSR00000074500	No	Microhomology
CC008	16	59,216,000	59,261,000	45	ND	ND	ND	PWK/PhJ	*Olfr199* (partial), *Olfr200-ps1*	ENSMUSR00000114105	Yes	
CC011	18	70,254,000	70,257,000	3	70,249,785	70,260,636	10,851	A/J	None	ENSMUSR00000378203	No	Unique
CC013	6	124,529,000	124,569,000	40	ND	ND	ND	PWK/PhJ to WSB/EiJ transition	*C1s1*	ENSMUSR00000235398, ENSMUSR00000439605, ENSMUSR00000235399, ENSMUSR00000439609, ENSMUSR00000439610	Yes	Complex SD
CC025	12	11,617,000	11,621,000	4	11,613,044	11,625,412	12,368	PWK/PhJ	None	None	Yes	
CC026	17	57,161,000	57,245,000	80	57,148,212	57,248,753	100,541	C57BL/6J	*Cd70*, *Tnsf14*, *C3*, *mir6978*, *Gpr108*	Many	Yes	Microhomology
CC030	8	43,624,000	43,626,000	2	ND	ND	ND	PWK/PhJ	*Gm5346* (partial)	None	Yes	
CC038	2	86,260,000	86,266,000	6	ND	ND	ND	CAST/EiJ	None	None	Yes	Olfactory receptor cluster
CC043	4	90,613,000	90,621,000	8	ND	ND	ND	NOD/ShiLtJ	None	None	No	
CC046	5	151,538,000	151,547,000	9	ND	ND	ND	CAST/EiJ	*Vmn2r-ps26* (partial)	None	Yes	
CC055	3	132,902,000	132,939,000	37	132,897,360	132,944,510	47,150	129S1/SvImj	*Npnt* (partial)	Many	No	Microhomology
CC056	8	55,080,000	55,094,000	14	ND	ND	ND	PWK/PhJ	*Gm8734*	None	Yes	
CC057	16	70,377,000	70,378,000	1	70,377,000	70,392,212	15,212	C57BL/6J	*Gbe1* (intron)	None	Yes	Proximal end overlaps with repeat
CC072	6	11,664,000	11,738,000	74	ND	ND	ND	WSB/EiJ	None	ENSMUSR00000431623, ENSMUSR00000222992, ENSMUSR00000222993,	Yes	
CC072	15	40,549,000	40,551,000	2	40,544,598	40,555,614	11,016	129S1/SvImj	None	None	No	Microsatellite
CC074	13	24,536,000	24,539,000	3	24,529,800	24,543,000	13,200	PWK/PhJ	None	ENSMUSR00000337301, ENSMUSR00000070027	Yes	Ends overlap with repeats. No exact breakpoints determined
CC074	13	27,113,000	27,123,000	10	ND	ND	ND	PWK/PhJ	*Prl3d2* (partial)	None	Yes	
CC074	13	33,303,000	33,304,000	1	ND	ND	ND	PWK/PhJ	None	None	Yes	
CC075	4	112,022,000	112,031,000	9	ND	ND	ND	WSB/EiJ	*Skint1* (partial)	None	Yes	
CC075	9	27,720,000	27,731,000	11	ND	ND	ND	CAST/EiJ	None	ENSMUSR00000462205	No	

The table provides the strain and the start and positions of the initial discovery step and the refinement step by BWT when applicable. It also indicates the founder haplotype, gene and regulatory content as obtained from Ensembl (v87). Finally, the table indicates whether the deletion overlaps with a known structural variant (SV) and defining characteristics. ND, not determined.

We designed PCR assays that discriminate between the presence and absence of the new structural variant for three deletions on chromosomes *3*, *13*, and *17* in CC055/TauUnc, CC007/Unc, and CC026/GeniUnc, respectively. We used these assays to validate the deletion in the corresponding sequenced sample and test its status in the MRCAs and in a representative cohort of current mice from the corresponding CC strain. The CC007/Unc deletion was fixed in both MRCAs and the current population. The deletion in CC026/GeniUnc was segregating in the MRCAs but fixed in the current population. This is noteworthy as this deletion removes the entire coding region of several genes, including the *complement factor 3* gene (*C3*) (Figure 6). Finally, the CC055/TauUnc deletion was segregating both in the MRCAs and in the UNC Systems Genetics Core Facility colony. This deletion removes eight coding exons of the *Npnt* gene (Figure 6 and Figure S7). To test the effect of the deletion on *Npnt* gene expression, we used brain tissue samples collected by our group as part of a survey CC population. *Npnt* is expressed in the brain of several of the CC founder strains (http://csbio.unc.edu/gecco/) (Crowley *et al.* 2015). We used two qPCR assays to measure *Npnt* messenger RNA (mRNA) levels, one assay targets the *Npnt* transcript outside of the deletion and one targets the *Npnt* transcript inside of the deletion region (Figure S7 and see *Materials and Methods*). For the assay within the deleted exons, CC055/TauUnc mice homozygous for the partial *Npnt* deletion had no gene expression, while the level of *Npnt* gene expression is negatively correlated with the number of deletion alleles in heterozygous and homozygous mice for the wild-type allele (*P* = 0.011; Figure S7). Furthermore, consistent with the prediction that the deletion does not alter the open reading frame of the *Npnt* transcript, the assay outside the deleted region amplified transcripts from RNA isolated from all three genotypes (Figure S7). The results suggest a linear relationship between average steady-state mRNA levels and the number of wild-type alleles. We hypothesize that the deletion either removes a regulatory element or leads to increased levels of mRNA degradation.

### Mapping of unplaced contigs of the mouse genome

**Figure 7:**
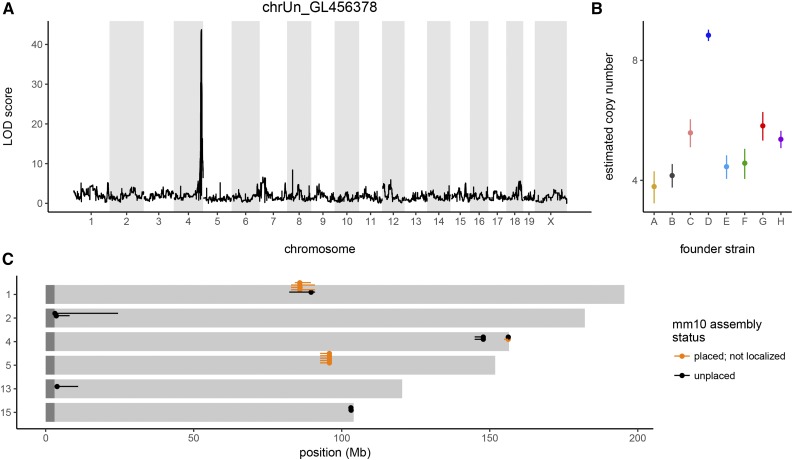
Mapping of unplaced sequences in the CC. (A) QTL scan demonstrating successful localization of GL456378, a contig not localized in the current mouse reference genome (mm10/GRCm38.p5), to distal chromosome *4*. (B) Estimated copy number of *GL456378* in founder strains. (C) Genomic distribution of 19 sequences localized using the CC. Gold, sequences previously assigned to a chromosome but not a specific position; black, sequences whose position was previously unknown. Dot indicates marker with maximum LOD score and line segment indicates 95% credible interval.

The current mouse reference genome assembly (mm10/GRCm38.p5) consists of 22 chromosome-level components (*1–19*, *X*, *Y*, and *M*) and 44 unplaced components composed almost entirely of repetitive sequences. We exploited the fact that repetitive sequences are often copy number variable to map 19 of 44 previously unplaced assembly components in the CC. These 19 sequences correspond to seven discreet loci across six different chromosomes (Figure 7 and Table S3). Mapping resolution of the unplaced contigs is relatively high, given the small size of the CC sequenced population (median resolution is 3.8 Mb and the range is 59 kb–21 Mb) but substantially lower than in the DO (Svenson *et al.* 2012). This is in line with expectation for Mendelian traits.

## Discussion

The concept of “genotype once phenotype many times” underlies the popularity of recombinant inbred panels (Silver 1995). In mouse, characterization of the genomes of such panels has improved significantly thanks to the use of more dense and informative genotyping arrays and recently with the use of affordable and robust whole genome sequencing technology. In both cases, the goal is to describe every recombinant inbred strain as a mosaic of haplotypes from the founder strains, with every region of the genome properly tracked and recombination breakpoints between haplotypes resolved to the closest pair of informative markers. Recent experience with mouse inbred strains indicates that genome characterization and annotation should be an ongoing community effort (Keane *et al.* 2011; Yalcin *et al.* 2011; Wong *et al.* 2012; Doran *et al.* 2016; Morgan *et al.* 2016b; Oreper *et al.* 2017).

Although *de novo* genome assembly should be the ultimate goal for all laboratory inbred strains, the two resources presented here, sequenced genomes and MRCAs, offer an excellent approximation to the true genome and have complementary strengths and uses. Both descriptions of the genomes are highly concordant, but researchers who have used the CC population prior to the date of birth of the sequenced sample and are performing genetic mapping should use the MRCAs, as they provide a more conservative view of the founder haplotypes that can be present in a given mouse from a given strain. MRCAs, as their name indicates, are ancestors of all CC mice distributed from that strain to the community. The haplotype reconstruction algorithm applied to the MRCAs is designed to retain any haplotype present among the MRCAs independent of its frequency or zygosity status. In contrast, the sequenced genomes represent just a single sample of two chromosomes—a diploid “snapshot”—from the CC colony. We are committed to increase the value of the sequenced data by genotyping additional contemporaneous mice and integrating both data sets to identify all regions of residual heterozygosity. We propose to use the first iteration MUGA array as the genotyping platform because although this array is lower resolution than more recent iterations, it was custom designed for the CC and may provide a cost effective platform to complete our goals. As a proof of concept, we genotyped three females from CC018/Unc and identified two additional regions spanning 23 Mb that were segregating in the CC018/Unc colony but fixed in the sequenced sample (Figure S8). We will expand this approach to all CC strains in the near future and make these results public as part of our ongoing efforts to characterize the genome of the CC with increased resolution and accuracy.

Potential users of the CC should be aware that although sequenced mice from most strains are considerably more recent in time than MRCAs, a similar amount of time and number of generations have elapsed since the birth of the sequenced samples and the present. Therefore, in the absence of increased selection (see below) today’s CC users should expect to see a similar amount of reduction in residual heterozygosity (Welsh and McMillan 2012).

Sequenced genomes provide a wealth of information absent from the MRCAs including refined recombination breakpoints (Figure 2 and Figure S2), founder haplotypes on the mitochondria and chromosome *Y* (Table 1), and a whole set of new variants arising by drift (Figure 5). Each one of these might have phenotypic consequences. For example, the chromosome *Y* from CAST/EiJ contains sequence present also on the *X* chromosome of other strains, increasing the dosage of a gene essential for early development (White *et al.* 2012; Chesler *et al.* 2016). Recombination can resolve whether coding and regulatory variants are inherited from one or another founder or represent a new mosaic combination (Figure 2). Finally, some of the *de novo* mutations such as large deletions should lead to null alleles in genes of well-known function. Alternatively, they can delete regulatory elements (Yue *et al.* 2014) and therefore provide a means to test their phenotypic effect. The CC is a mapping population with high levels of genetic diversity. An example of its value is the mapping of unplaced contigs, which improves the accuracy of the mouse reference genome.

Founder haplotype assignment based on whole genome sequences still poses considerable challenges in the presence of structural variation. In this initial analysis, we decided to mask these regions to avoid introducing systematic errors in the haplotype reconstruction and mutation detection due to paralogous variation (Morgan *et al.* 2016b). We believe that this has limited effect on the haplotype assignment because these regions are cold spots for recombination (Morgan *et al.* 2017) and thus flanking regions tend to be concordant. Regions of recent IBD between the five classical inbred founder strains of the CC, which cover 13% (IBD between all five) to 92% (IBD between any two) of the genome (Yang *et al.* 2011), pose an additional challenge. By definition, informative variants are few and far between in IBD regions, imposing a fundamental limit on the accuracy of haplotype reconstruction. Incorporation of new haplotype-level variants discovered in the CC population and tuning of the HMM in these regions may help mitigate these issues, but absolute certainty is unlikely to be achieved for all of them. This difficulty is less pronounced in MPP from other species, whose founders tend to have less convoluted ancestry (Kover *et al.* 2009; Huang *et al.* 2011; King *et al.* 2012).

Two related mouse genetic reference populations, the CC and the DO, were designed with the expectation that selection would have a modest effect on their genomes. Based on the massive extinction rate among CC funnels (Shorter *et al.* 2017), it is obvious that selection has played a major role in shaping the genomes of the CC strains. Here we show that the contribution of CAST/EiJ and PWK/PhJ is significantly below the expectations in the autosomes of the 56 CC strains with contributions from all eight founder strains. This systematic underrepresentation cannot be fully ascribed to drift. Interestingly, the founder contribution in the first generation of inbreeding (Liu *et al.* 2014), in the pre-CC experiments (Aylor *et al.* 2011), and in the initial description of the genomes (Collaborative Cross Consortium 2012) was balanced (Figure S9). Furthermore, in 347 extinct funnels reported in a companion manuscript (Shorter *et al.* 2017), the autosomal haplotype frequencies are very close to the input. We conclude that genome-wide selection against haplotypes from the two wild-derived strains from subspecies underrepresented in the genome of the founders of the CC is responsible for these observations. Inspection of the haplotype frequencies across the genome (Figure S5), the reduced contribution to many autosomes (Figure S6), and the unexpected bias in founder contribution to the residual heterozygosity strongly suggests that selection takes the form of epistatic interactions at multiple loci (Dobzhansky 1934, 1936). We hypothesize that similar epistatic combinations of alleles from different mouse subspecies are not only responsible for extinction in the CC but also for the prevalence of strains with extreme or emerging phenotypes (Rasmussen *et al.* 2014; Rogala *et al.* 2014). In other words, the expansion of the phenotypic range in the live CC strains is the positive side to the astounding rates of extinction observed in the project as a whole. If this is the case, then extinct funnels may provide valuable clues to interpret the results in distributable CC strains. Inspection of the founder contribution to each individual strain in the strains with eight founders indicates that selection against *castaneus* and *musculus* haplotypes operated in most strains (Figure S1). In fact, in strains with fewer than eight founders but with contributions of CAST/EiJ and/or PWK/PhJ, the deficit in the contribution of these strains is magnified although it does not reach statistical significance.

In addition, there is obvious selection on chromosome *X* as expected from hybrid sterility studies in mice (Forejt and Iványi 1974; Oka *et al.* 2004; Payseur *et al.* 2004; Storchová *et al.* 2004; Good *et al.* 2008; Turner *et al.* 2014; Balcova *et al.* 2016) and potentially on chromosome *Y* against the WSB/EiJ strain. Finally, there is an unexpected deficit of live strains with CAST/EiJ and PWK/PhJ mitochondria. These two founder strains were well represented among the 701 funnels started at ORNL (11.55 and 14.12% of funnels had CAST/EiJ and PWK/PhJ as donors of the mitochondrial genome, respectively) (Shorter *et al.* 2017) so we suspect that these deficits reflect the action of true mitonuclear incompatibilities.

Surprisingly, live CC strains do not have distortion at the *R2d2* locus, despite the fact that transmission ratio distortion in favor of the WSB/EiJ allele has been reported at the locus in the pre-CC experiment (Aylor *et al.* 2011) and in the initial description of the genomes (Collaborative Cross Consortium 2012), and that selective sweep almost wiped out genetic variation for a large section of chromosome *2* in the DO population (Chesler *et al.* 2016; Didion *et al.* 2016). Transmission ratio distortion (TRD) at *R2d2* is caused by meiotic drive associated with a large copy number gain but requires the action of unlinked modifiers (Didion *et al.* 2015). Perhaps driver alleles were purged from the live CC strains early enough to avoid TRD (Didion *et al.* 2016). Although we purged the causative allele at *R2d2* from the DO population (Chesler *et al.* 2016), there is no guarantee that further unexpected forms of selection may affect both the CC and the DO populations.

The benefits of inbred strains in experimental research derives from their long-term genetic stability, which helps to ensure reproducibility of findings, allows increased precision of phenotypes by measurement of multiple genetically identical animals, enables powerful comparison of treatment effects within a constant genetic background, and enables the integration of data collected across multiple labs and at different times. However, the ideal of absolute genetic stability cannot be achieved. The inexorable forces of mutation and genetic drift will result in a slow but steady accumulation of new mutations, some of which will have phenotypic consequences. The rate of genetic drift will depend on the effective population size and thus on the breeding and colony-management practices. Breeding strategies that employ cryopreservation to minimize drift can only be partially effective and may themselves result in new, unexpected sources of variation (Bouquet *et al.* 1993).

With the advent of inexpensive whole genome sequencing, it has become feasible to monitor and precisely quantify the rate and impact of genetic drift. In our analysis of 69 inbred CC strains, we were able to identify and place a lower bound on the rate of fixation of new SNPs and small indel variants. These rates are dependent on our current colony management practices but it is not likely that we can reduce drift to much lower levels. While small in number, the changes are cumulative. The great majority of new variants will be phenotypically neutral but some will have phenotypic consequences that may range from subtle to dramatic. Because new mutations are specific to one strain, they cannot be mapped directly using the strain panel, but they can be localized using secondary crosses. Rather than focus on the negative impacts of new mutations, we would like to emphasize their potential for providing new models for disease-related phenotypes. Identification of mutations and their likely functional consequences is key to leveraging genetic drift to our advantage. This is exemplified by studies in which crosses between very closely related strains have led directly to the identification of causal polymorphisms (Kumar *et al.* 2013).

We have identified a number of large deletions (10 kb or greater) that have arisen in individual CC strains. Large deletions are present in approximately one-third of the sequenced strains and are likely to impact coding genes and functional elements in the genome, often resulting in loss of function. *Npnt* provides an example of a deletion with a likely functional effect on gene function, despite the fact that the deletion is only partial and the open reading frame remains unaffected. *Npnt* encodes the extracellular matrix nephronectin protein that promotes kidney development. Mice lacking the *Npnt* gene show kidney agenesis due to its role in ureteric bud invasion and branching during kidney development (Linton *et al.* 2007). The deletion in the CC055/TauUnc strain appears to be associated with a mild reduction in gene expression (Figure S7), but most importantly, it also deletes the majority of the annotated domains in the NPNT protein (Table S7).

Strains carrying specific and well-characterized deletions present unique and powerful tools for interrogating the function of elements in the deleted regions. While we have not pursued the characterization of other structural variants at this time, the potential for deriving functional insights from copy number gains, inversions, and translocations is promising.

The impact of genetic drift on an inbred strain panel is fundamentally different from its impact on an outbred population. Changes that accumulate and fix in inbred strains are (relatively) stable and are restricted to the strain in which they arise. In an outbred resource population, there will be a constant turnover of new variants, most often resulting in their loss from the population. However, in some cases, drift or selection could potentially lead to fixation of new variants with a consequent loss of standing variation in the outbred resource (Chesler *et al.* 2016; Didion *et al.* 2016) and affect genetic networks through altered interactions (Tyler *et al.* 2017). Monitoring of inbred *vs.* outbred genetic resource populations will require different approaches but is important for both ensuring the integrity of the resources and as an opportunity for new discoveries.

## Supplementary Material

Supplemental material is available online at www.genetics.org/lookup/suppl/doi:10.1534/genetics.116.198838/-/DC1.
