# Supplementary material for: Genomes of the Mouse Collaborative Cross
Source: Genetics. 2017 Jun 6;206(2):537–56. doi: 10.1534/genetics.116.198838 (PMC5499171; doi:10.1534/genetics.116.198838)
Supplement: Supplementary file 1 [file 537Data_documentation.docx]

**Genomes of the Mouse Collaborative Cross**

Anuj Srivastava, Andrew P Morgan, Maya L Najarian, Vishal Kumar Sarsani, J Sebastian Sigmon, John R Shorter, Anwica Kashfeen, Rachel C McMullan, Lucy H Williams, Paola Guisti-Rodríguez, Martin T Ferris, Patrick Sullivan, Pablo Hock, Darla R Miller, Timothy A Bell, Leonard McMillan, Gary A Churchill, Fernando Pardo-Manuel de Villena

**Genetic Material**

Mice from the Collaborative Cross can be purchased from the UNC Systems Genetics Core Facility website <http://csbio.unc.edu/CCstatus/index.py>,

The nomenclature of the CC can be found in the results section of this manuscript, and in the 2012 Collaborative Cross Consortium paper “The Genome Architecture of the Collaborative Cross Mouse Genetic Reference Population” (Collaborative Cross Consortium, 2012 GENETICS 190**:** 389-401):

*“Once a strain is deemed distributable it is renamed as CCxxx/yyy were “x” are consecutive numbers and “y” are several letter identifiers of the breeding site where the CC strain was initiated and currently maintained (Unc, Tau and Geni).”* Srivastava *et al*. 2017.

“*During the generation of the CC population, CC-UNC lines are named with the prefix OR (that stands for the two first letters of the Oak Ridge National Laboratory) followed by a number with two to four digits. CC-TAU lines are named IL (which represents the first two letters of the International Livestock Research Institute) followed by a number with two to four digits. CC-GND lines have unique names followed by a two-letter code reflecting the strain located in positions 1 and 8 of the funnel (Figure 1 and also see Chesler et al. 2008; Aylor et al. 2011; Threadgill et al. 2011). Once the CC lines are deemed complete (>97% inbred), they will be renamed in accordance with the rules of the International Nomenclature Committee (see Discussion). Specifically, each line will be named CC#/@, where # are four digits from a consecutive sequence across all three CC populations and @ is the location from whence the line originated (Unc, US lines; Tau, Israeli lines; and Geni, Geniad lines). For example, the first completed line, OR867, is now CC0001/Unc and the second line, IL6211, is CC0002/Tau*.” CC Consortium 2012.

**Raw Genotype file**

The unfiltered genotype files for the 69 sequences samples genotyped in GigaMUGA, 69 MRCAs that are genotyped in MegaMUGA, and the 3 samples from CC018/Unc genotyped in MUGA can be found in three different repositories: <https://www.med.unc.edu/mmrrc/genotypes>, <csbio.unc.edu/CCstatus/CCGenomes> and [https://doi.org/10.5281/zenodo.377036](https://doi.org/10.5281/zenodo.377036).

The 3 platforms, GigaMUGA, MegaMUGA, and MUGA have different markers between them, but within each platform markers are consistent. The nomenclature and design for these platforms is explained in Morgan *et al*. 2016. The Mouse Universal Genotyping Array: From Substrains to Subspecies. G3 https://doi.org/10.1534/g3.115.022087

**Results file**

There are several different files derived from the sequence data uploaded on two repositories that contain results information. Additionally, results from the unplaced contigs can be found in Table S3. “Mapping of unplaced contigs”. Results from SNPs and Indel analysis can be found in Table S5. “High Impact SNP and indel variants” and Table S6 “Frequency of private SNPs and Indels by Haplotype”.

The ENA accession PRJEB14673 provides access to the following files:

- CC_69_Samples_vcf_merge.db: SQLlite database file

- Genomestrip_raw.vcf.gz: genome strip unfiltered calls

- Joint_69_flagged.tab: VCFfile obtained by  joint haplotype variant caller

- Merged_69_flagged.tab: VCF file merged from single sample calling

- pseudoGenomes.tar.gz: Pseudo genome files

Zenodo accession #377036 ([https://doi.org/10.5281/zenodo.377036](https://doi.org/10.5281/zenodo.377036)) provides access to the following files:

- fastq_filelist: list of fastq filenames deposited with ENA

- bam_file_list: list of bam filenames deposited with ENA

- CC_69_samples-1kb_haplo.bed: read-coverage in kb bins, used for deletion analysis

- bin_creator.py: code to create read depth files

- count_calculator.sh: shell script to create read depth files

- CNV_Analysis_1k.R: analysis of read depth data

- CCStrains.csv: summary information about CC strains

- Private_Variants.csv: list of 28000 private variants

- PrivateVariants.R: analysis of private variants data

- Hap files based on GigaMUGA genotypes, whole genome sequence, and MRCAs

- 36 state probabilities for 69 MRCAs
