# Supplementary figures and images for: Genomes of the Mouse Collaborative Cross
Source: Genetics. 2017 Jun 6;206(2):537–56. doi: 10.1534/genetics.116.198838 (PMC5499171; doi:10.1534/genetics.116.198838)

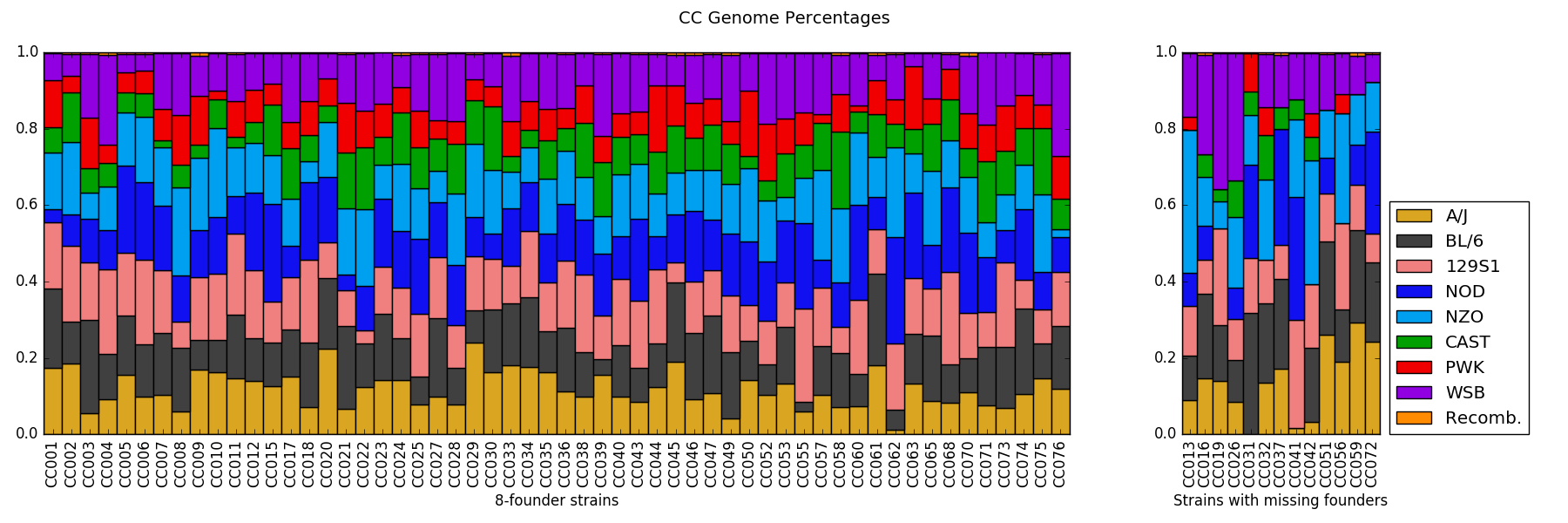

Supplement: Supplementary file 2 [file 537FigureS1.png]

cumulative proportion

autosomes

chromosome X

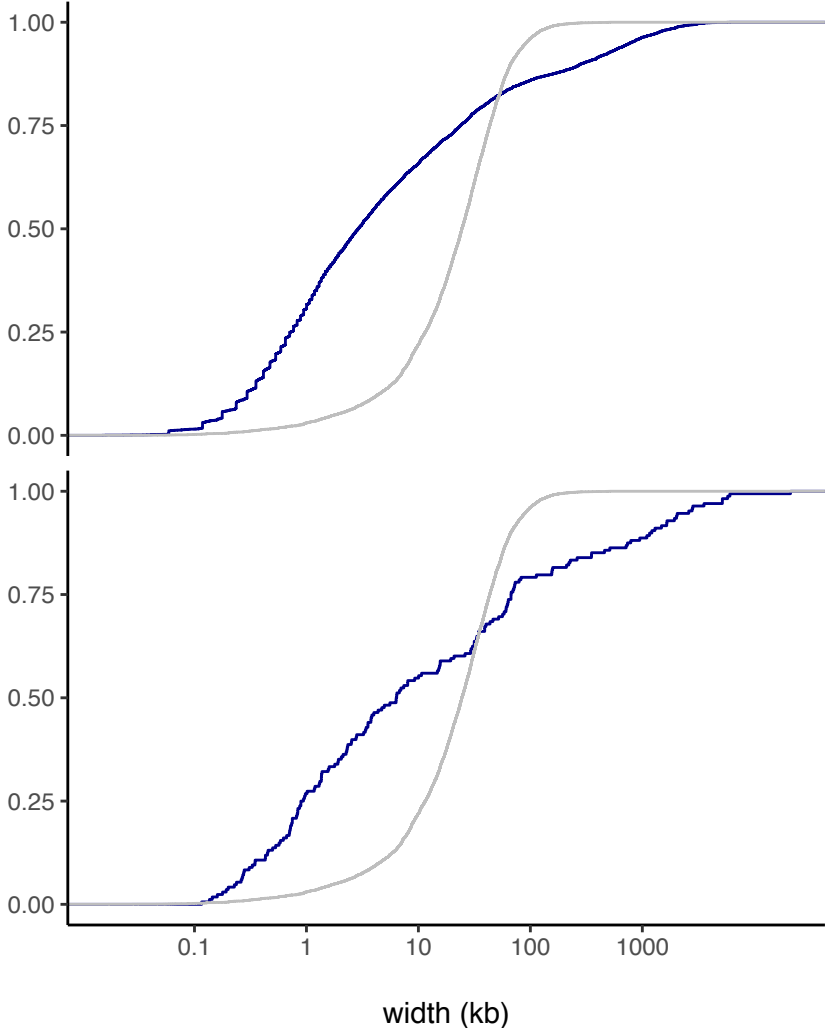

refinement — 77K array — whole-genome sequence

Supplement: Supplementary file 3 [file 537FigureS2.pdf]

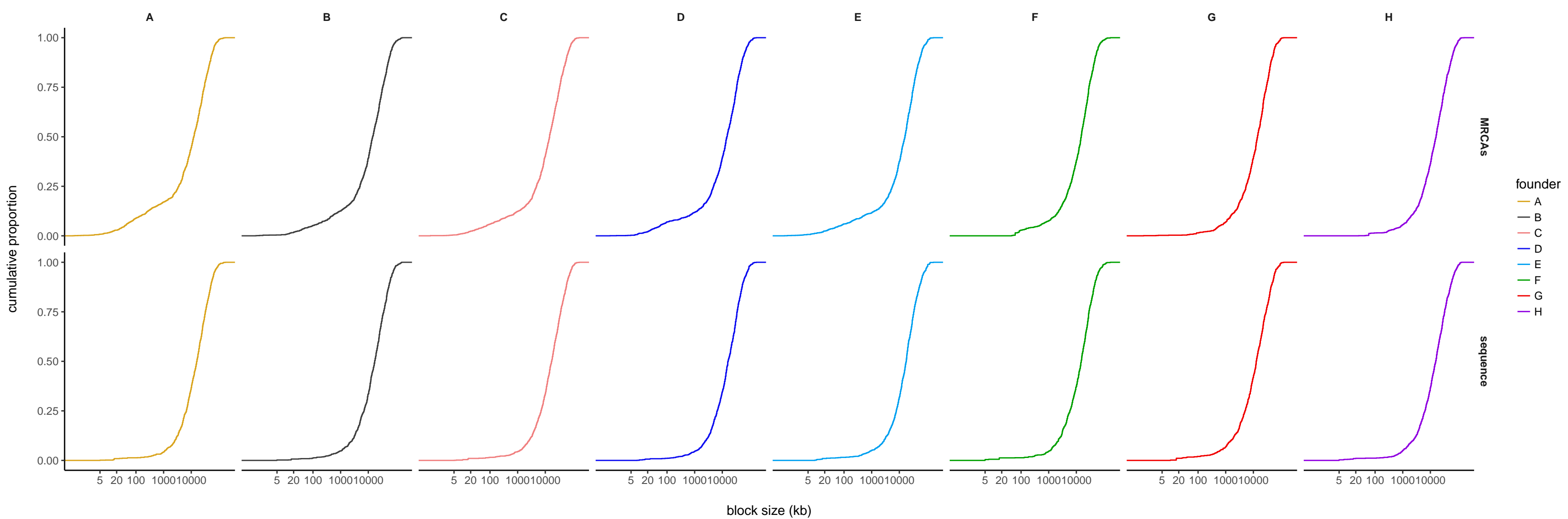

Supplement: Supplementary file 4 [file 537FigureS3.pdf]

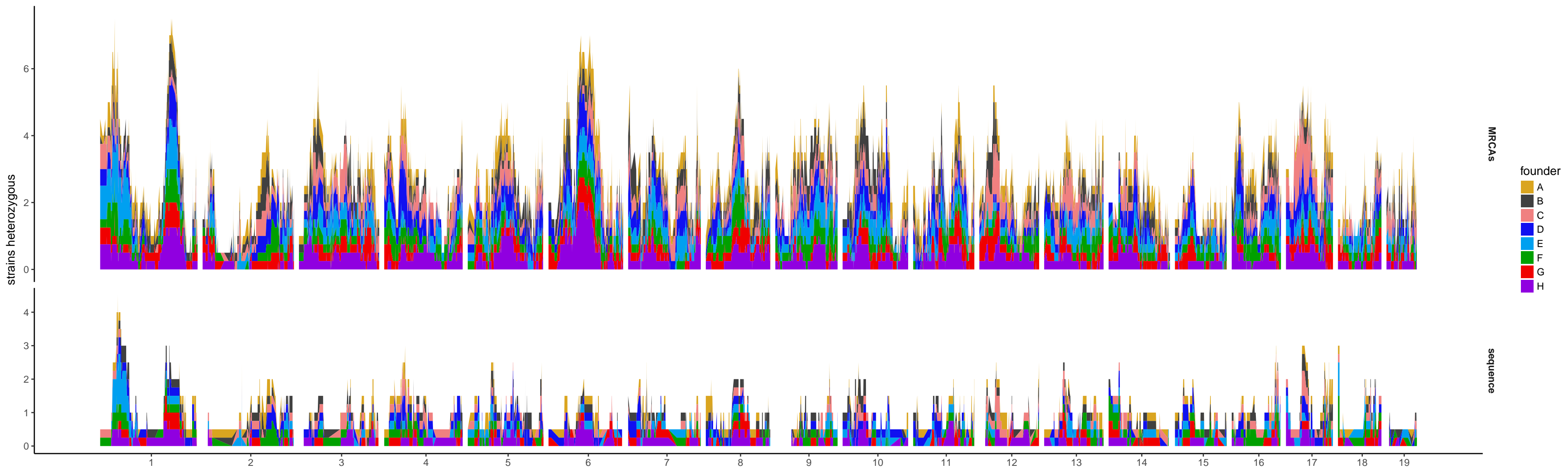

Supplement: Supplementary file 5 [file 537FigureS4.pdf]

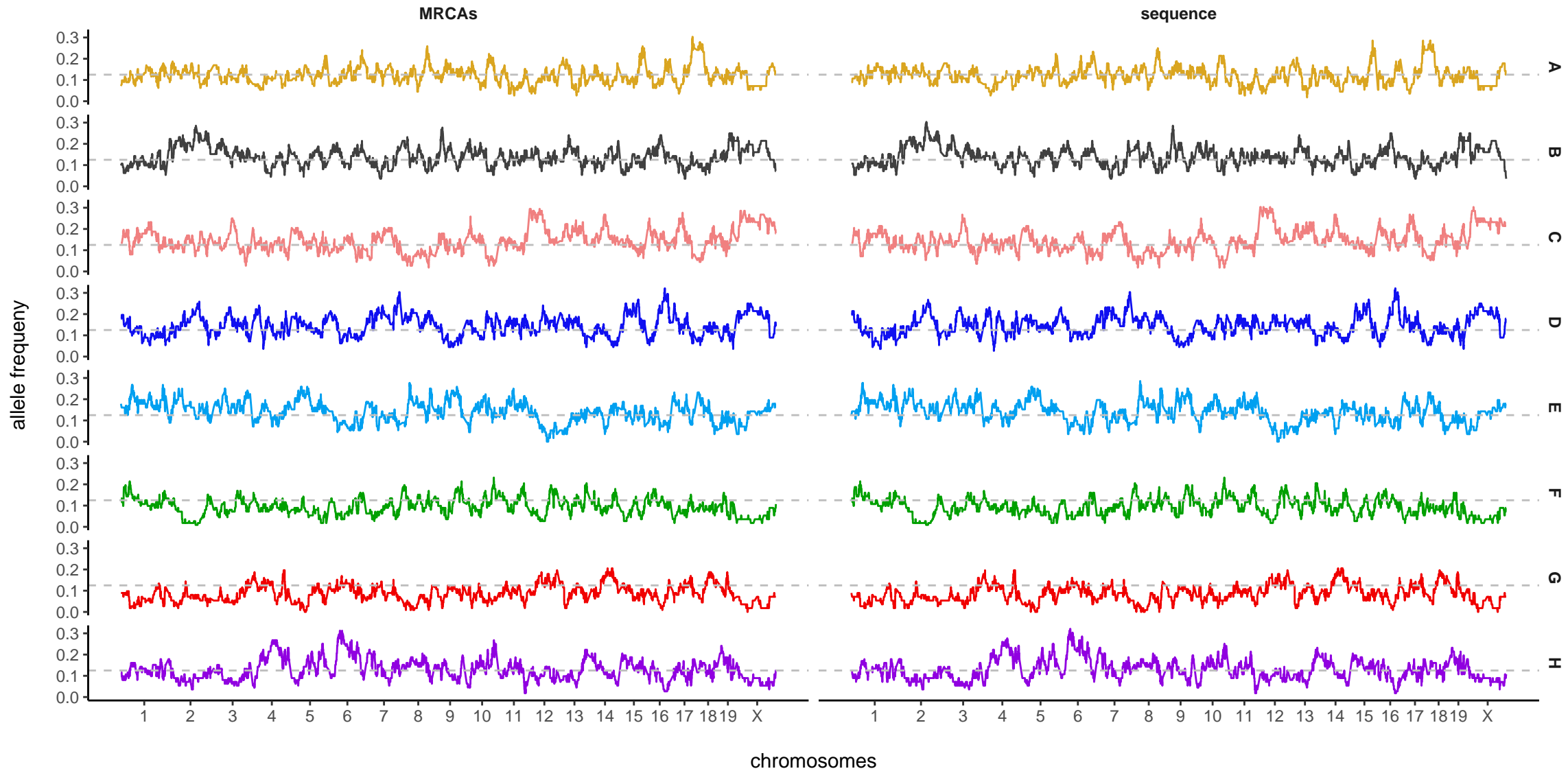

Supplement: Supplementary file 6 [file 537FigureS5.pdf]

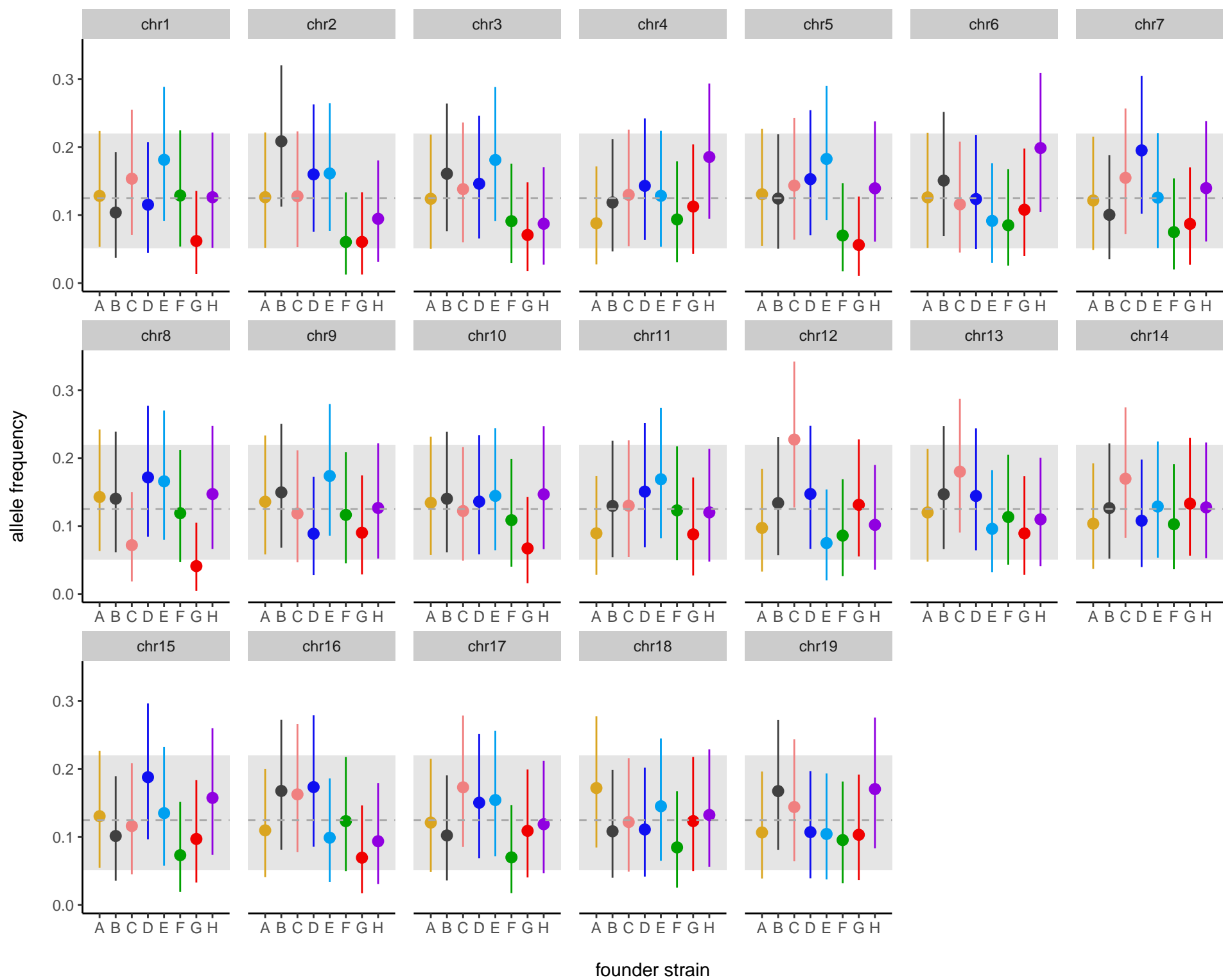

Supplement: Supplementary file 7 [file 537FigureS6.pdf]

**A**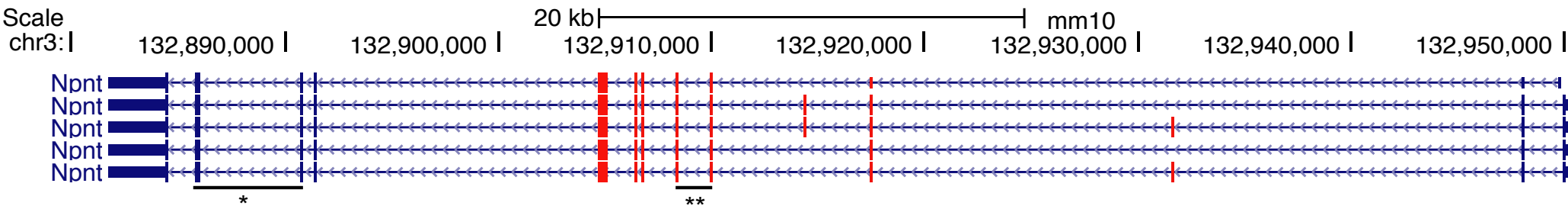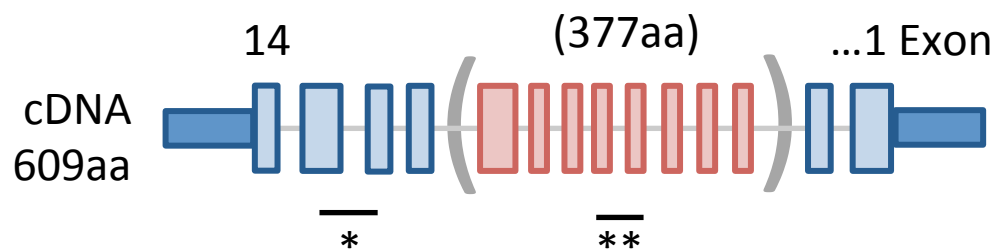**B**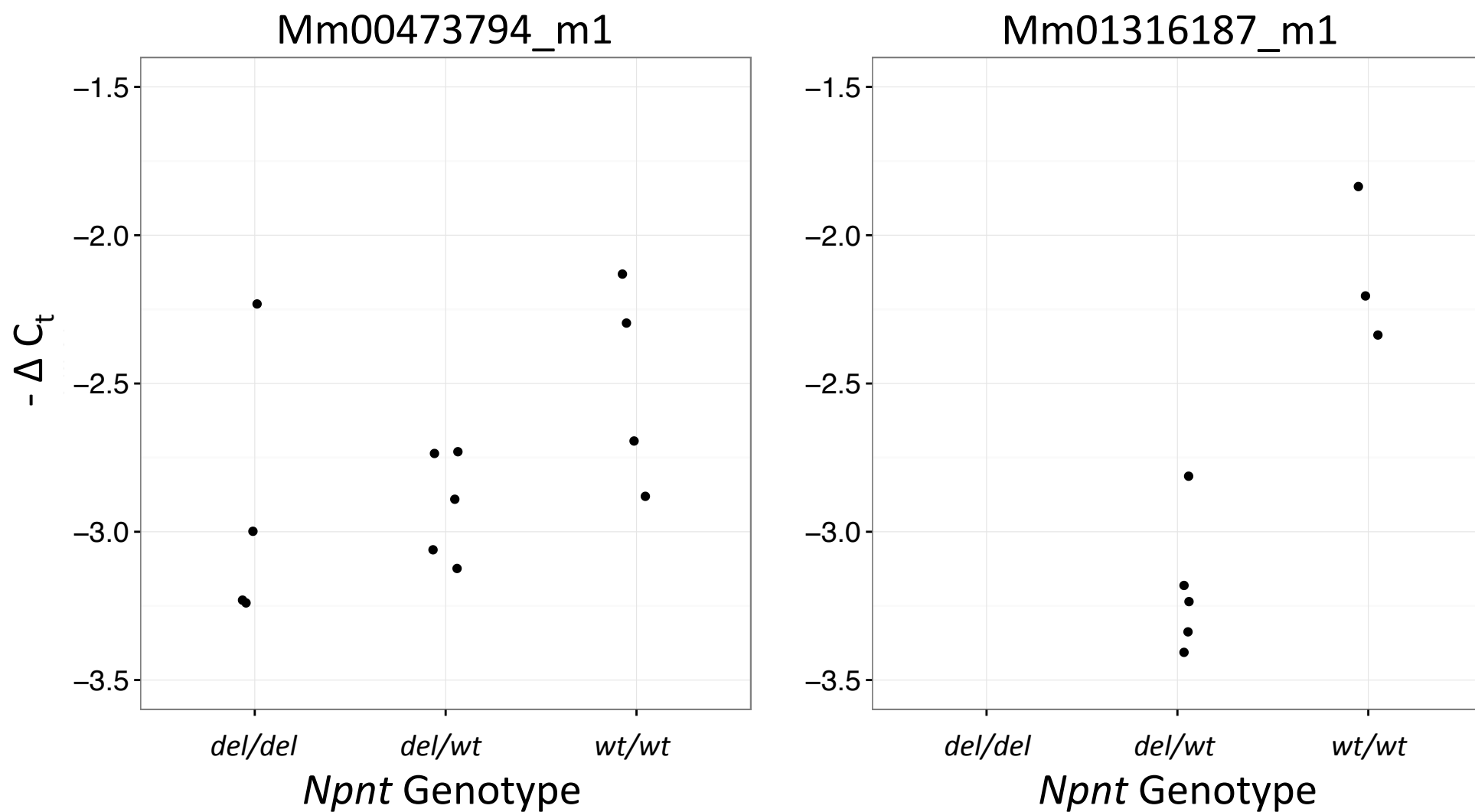

Supplement: Supplementary file 8 [file 537FigureS7.pdf]

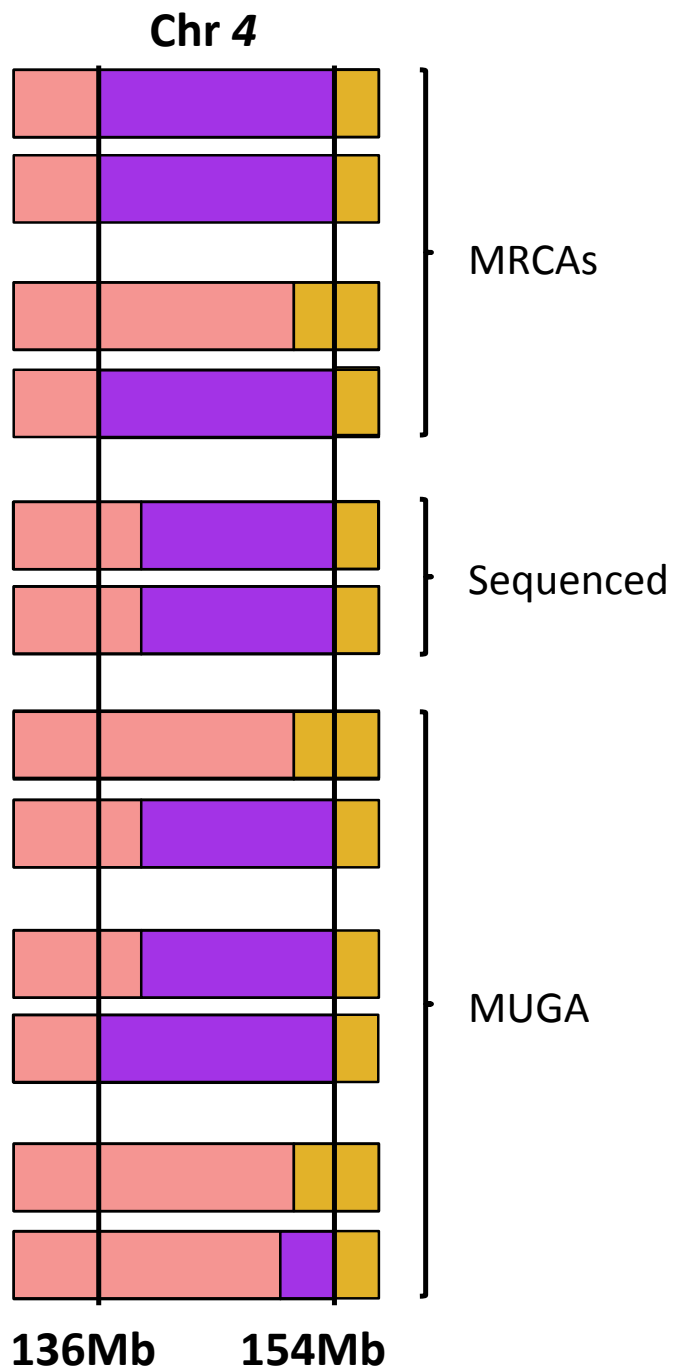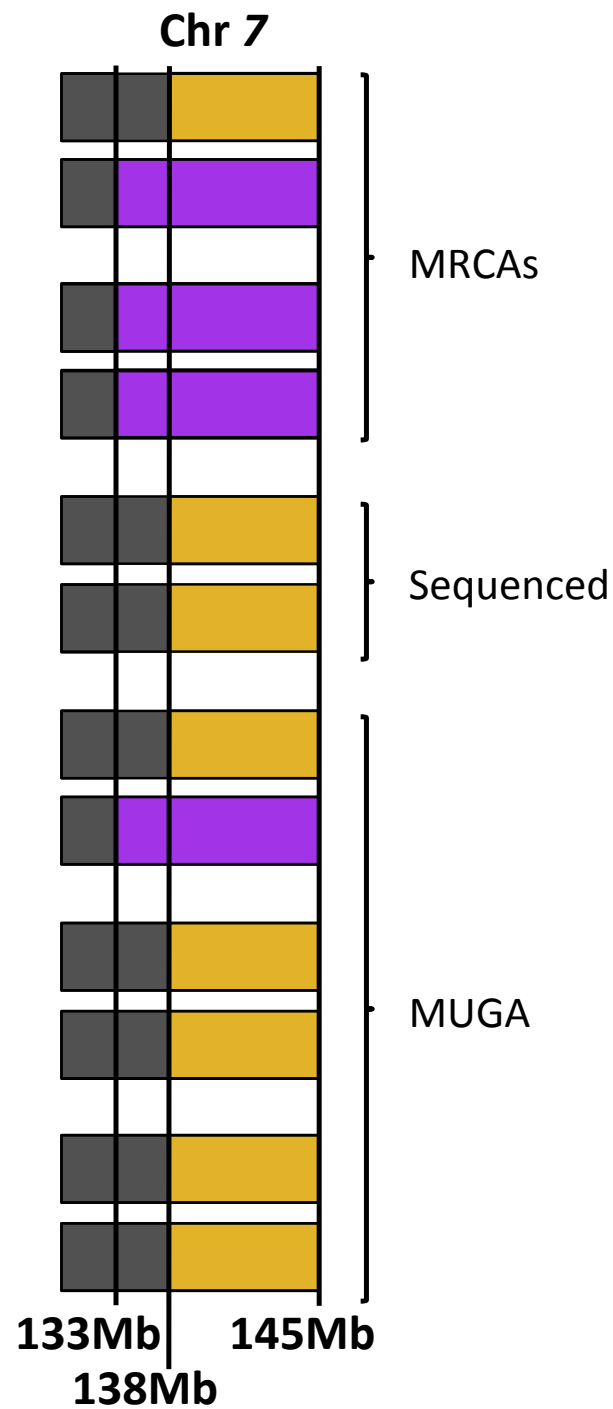

Supplement: Supplementary file 9 [file 537FigureS8.pdf]

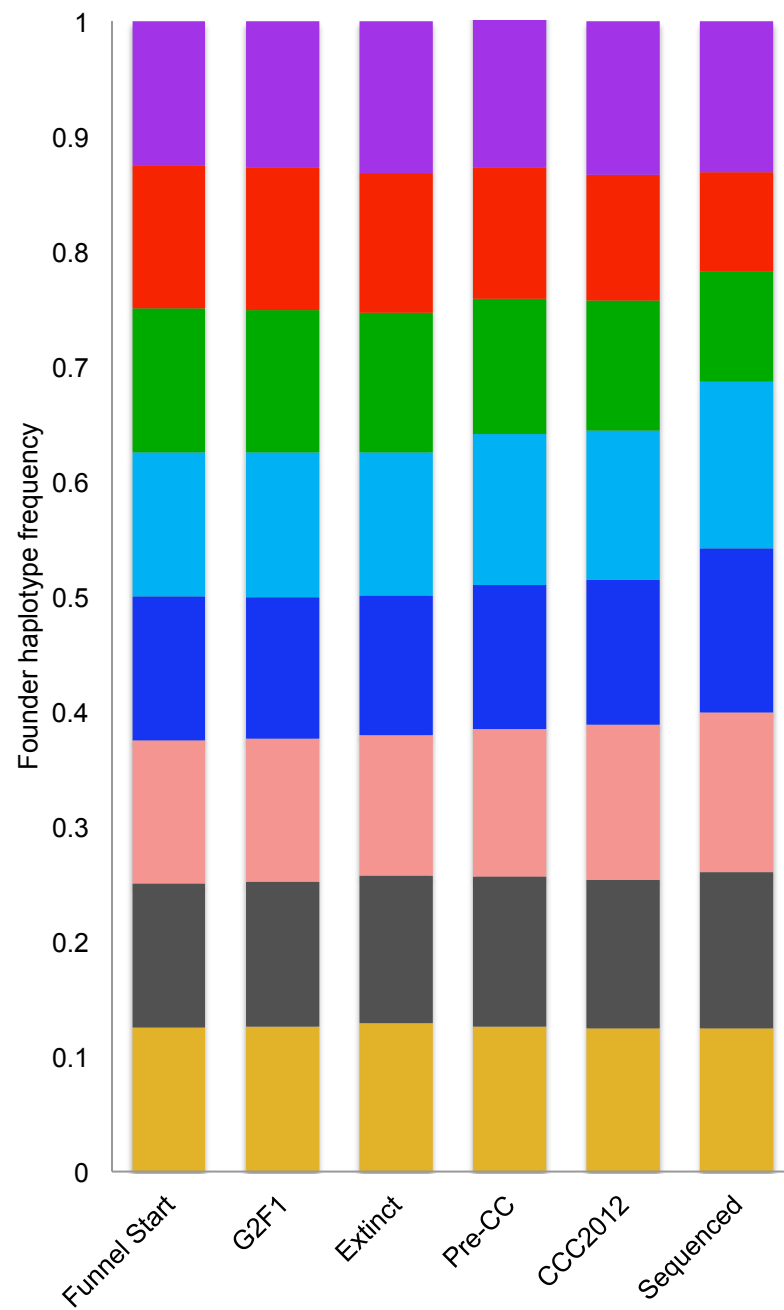

Supplement: Supplementary file 10 [file 537FigureS9.pdf]
